# Toward comprehensive multimodal neuromonitoring of surrogates of cerebral autoregulation in traumatic brain injury: insights from a hyperventilation protocol

**DOI:** 10.1117/1.NPh.13.3.035010

**Published:** 2026-09-17

**Authors:** Susanna Tagliabue, Maria Martorell Ruiz, Jonas B. Fischer, Federica Maruccia, Anna Rey-Perez, Lourdes Exposito, Marcelino Baguena, Jana M. Kainerstorfer, Juan Sahuquillo, Maria Antonia Poca, Turgut Durduran

**Affiliations:** a ICFO-Institut de Ciències Fotòniques, The Barcelona Institute of Science and Technology, Barcelona, Spain; b Neurotraumatology and Neurosurgery Research Unit (UNINN), Vall d’Hebron Research Institute (VHIR), Barcelona, Spain; c Vall d’Hebron University Hospital, Neurotrauma Intensive Care Unit, Barcelona, Spain; d Carnegie Mellon University, Department of Biomedical Engineering, Pittsburgh, Pennsylvania, United States; e Vall d’Hebron University Hospital, Department of Neurosurgery, Barcelona, Spain; f Universitat Autònoma de Barcelona, Barcelona, Spain; g Institució Catalana de Recerca i Estudis Avançats (ICREA), Barcelona, Spain

**Keywords:** cerebral autoregulation, multimodal neuromonitoring, hybrid diffuse optics, traumatic brain injury

## Abstract

**Significance:**

It is hypothesized that neurocritical-care targeting cerebral autoregulation (CAR) would lead to improved outcomes. However, there is no single accepted CAR index due to limited accessibility of the methods and the complexity of interpretation. It is imperative to introduce accessible and direct methods.

**Aim:**

We aim to compare traditional CAR indices and those on hybrid near-infrared diffuse optical (DO) monitors on a clinical population undergoing a controlled intervention.

**Approach:**

Traumatic brain injury patients undergoing a hyperventilation protocol were monitored. Seven CAR indices based on the common correlation approach were derived. The detection of CAR alteration by hyperventilation, their variability, and the simultaneity of the detected changes were quantified.

**Results:**

Seventeen patients have been monitored, showing a predominant decrease in CAR due to hyperventilation in some but not all indices. The quantified variability has demonstrated that DO-based indices could be more sensitive to small changes, and a compound “agreement score” has indicated that most recordings (18/20) displayed directional consistency, supporting the robustness of multimodal assessment.

**Conclusions:**

We have demonstrated that different CAR indices lead to different interpretations and further analysis and clinical trials are merited. We call for the release of open data-sets to enable this.

## 1 Introduction

The bedside, continuous, and interpretable assessment of cerebral autoregulation (CAR) is widely suggested to play a pivotal role leading to the next-generation brain-oriented, individualized therapeutic decision-making and better outcomes.^1^^,^^2^ Unfortunately, despite decades of research and different clinical trials showing improved outcomes, a reliable, widely accessible, and interpretable evaluation of CAR remains elusive.^3^^–^^7^ In response to this challenge, the cerebrovascular research network (CARNet^8^) was established as an international, multidisciplinary initiative aimed at improving the reliability, comparability, and interpretation of CAR metrics. CARNet has published consensus white papers that summarize current methodologies and recommendations, which remain an active subject of discussion and refinement within the field.^3^^,^^4^ Within this evolving framework, it is imperative to test and validate a standardized method for quantifying CAR and, furthermore, to make it accessible for widespread clinical use.^3^^,^^4^^,^^9^^–^^11^

Originally described by Lassen in 1959,^12^^,^^13^ CAR is traditionally referred to as the brain’s ability to maintain stable cerebral blood flow (CBF) across a range of cerebral perfusion pressures (CPP). CPP is further defined as the difference between the mean arterial blood pressure (MABP) and the intracranial pressure (ICP), i.e., [Eq. (1)].^3^^,^^5^^,^^11^^,^^13^^–^^16^

CPP=MABP-ICP (1)

This definition has since been further modified to extend this traditional “static” CAR by a “dynamic” CAR (dCAR), reflecting how quickly and effectively cerebral vessels constrict or dilate to buffer short-timescale (over seconds or heartbeats) spontaneous fluctuations in CPP or MABP, thereby stabilizing CBF in the face of transient hemodynamic disturbances.^3^^,^^5^^,^^11^^,^^13^^,^^15^^–^^17^ It is assumed that CAR is impaired when compensatory responses are delayed or absent.^18^

Since Lassen’s original work, numerous invasive and noninvasive techniques have been utilized to assess dCAR. The most common method is a time-domain index, and the pressure reactivity index (PRx) is calculated by quantifying the correlation between slow-wave (10s of seconds to minutes) fluctuations in ICP and MABP with a moving window and Pearson’s correlation coefficient.^19^^,^^20^ It is widely used and has been shown to have potential clinical value in various clinical trials.^19^^–^^24^ PRx directly reflects cerebrovascular pressure reactivity, a fundamental component of dCAR, and enables continuous bedside monitoring. Its clinical prominence arises from extensive validation in traumatic brain injury (TBI) patients, where it has demonstrated potential prognostic and therapeutic.^19^ However, it is only applicable when invasive ICP monitoring is routinely practised, hence reducing its widespread use.^19^

Other time-domain methods calculate–here we focus on time-domain methods and leave emerging algorithms based on transfer-function analysis as the subject of future research. This choice was made since time-domain methods dominate the clinical literature–most commonly, a Pearson’s correlation coefficient between a pressure signal (i.e., MABP, ICP or CPP) and a CBF surrogate.^4^^,^^25^ Clinically, pressure signals are standardly obtained via invasive ICP monitors, arterial lines, or a noninvasive finger cuff blood pressure monitor. CBF surrogates include invasive methods (laser Doppler flowmetry, thermal diffusion), transcranial Doppler ultrasound (TCD) for macrovascular CBF,^26^ and near-infrared (NIR), diffuse optical techniques commonly quantifying cerebral oxygenation.^3^^,^^6^^,^^16^^,^^27^^–^^34^ Using the correlation approach (ranging from minus one to one), values near zero or negative imply preserved CAR, whereas values above 0.3 to 0.45 often suggest an impairment.^16^^,^^35^^–^^37^ However, this is highly dependent on the utilized modalities. This arises from differences in the underlying physical and physiological assumptions inherent to each modality and its respective signal-processing methods, which confounds cross-comparison between studies and patient populations.^1^^,^^2^^,^^38^ Therefore, no single CAR index has achieved consensus for routine use,^6^^,^^7^^,^^28^^,^^39^ and no gold standard has been established so far.^7^^,^^16^^,^^17^^,^^31^^,^^40^

In other words, as Brassard^17^ discusses, CAR remains a "black box" phenomenon in many respects, and a single-modality approach provides an incomplete and decontextualized view of the brain’s complex dynamic physiology. This can often be misleading, as a seemingly normal CAR measurement might fail to reveal critical issues like elevated intracranial pressure or poor brain tissue oxygenation and does not account for other factors that influence CAR. The emerging consensus is that multiple aspects of CAR should simultaneously be evaluated by a multimodal approach.^7^^,^^17^^,^^28^

Hybrid diffuse optical (DO) monitoring, i.e., the simultaneous use of noninvasive modalities based on NIR light that measures both cerebral oxygenation and blood flow, has been suggested as a new method to derive not only several components of CAR but also explain the sufficiency of tissue perfusion and oxygen consumption.^41^^,^^42^ In particular, we utilize a combination of time-resolved spectroscopy (TRS)^43^^,^^44^ and diffuse correlation spectroscopy (DCS).^45^^–^^47^ Although the use of this hybrid combination for continuous bedside monitoring has not been extensively demonstrated in TBI, validations in other neurologically ill populations have highlighted its potential to address existing challenges in CAR monitoring.

In this work, we have leveraged a previously acquired dataset from TBI patients undergoing a period of controlled, passive hyperventilation (HV) with hypocapnia^48^ to compute seven CAR indices (detailed in Sec. 2.5.1) across modalities and two brain hemispheres. This data set has been comprehensively vetted out for quality^49^ and contains longitudinal, synchronized data from both clinical and research modalities. Furthermore, it covers pre-, during, and post-hyperventilation periods, allowing for the evaluation of the response to this controlled intervention, which may induce a robust, transient change in CAR.^50^^–^^55^ Our overall aim is to explore differences in combinations of modalities in terms of the variability of the resulting CAR indices, in terms of the detection of a potential alteration of CAR due to the hyperventilation challenge, and to describe the limits and periods of agreement.

As such, this study presents one of the most comprehensive simultaneous assessments of CAR indices to date. By comparing invasive and noninvasive optical-derived surrogates, we aim to contribute to the interpretation of multimodal data and its use for CAR assessment.

### 1.1 Hypotheses

We have hypothesized that hybrid DO techniques offer a noninvasive approach to monitor CAR longitudinally, with performance quantitatively comparable to invasive and other differently derived existing methods.

We have further anticipated that CAR indices would exhibit significant and interpretable (increase versus decrease) changes in response to performing HV, consistent with prior studies suggesting improved CAR after the end of HV.^50^^–^^55^ Specifically, we have hypothesized that improvement in CAR would manifest as a decrease in correlation-based CAR indices after the end of HV.

We have also hypothesized that DO-based indices would demonstrate lower temporal variability than PRx, as quantified by standard deviation and interquartile range (IQR), thereby providing greater sensitivity to detect small but meaningful changes in CAR.

Finally, building upon recent suggestions^2^ that if multiple CAR indices indicate the same type of change (increase versus decrease), there is a higher confidence in interpreting the result as being better or worse, we have tabulated their numbers and directionality and developed an index based on their concordance.

## 2 Methods

In this retrospective study, we have used the same materials and methods as described in Ref. 48 for patient selection, protocol, and measurement instrumentation. Therefore, only a brief summary is provided here. The analytical methods used to test the hypotheses, instead, are original to this work.

### 2.1 Human Subjects

TBI patients admitted to the Neurotraumatology ICU at the Vall d’Hebron University Hospital have been included according to these criteria: (1) being older than 16-year-olds, (2) having a Glasgow coma scale (GCS) between 3 and 15 at admission, (3) needing invasive ICP monitoring and mechanical ventilation from the admission or in case of clinical deterioration, (4) being in normocapnia, defined as having an arterial partial pressure of carbon dioxide (${\mathrm{PaCO}}_{2}$) between 35 and 40 mmHg, at the moment of the enrollment, (5) being in a sufficiently stable cerebral hemodynamic state that allowed for a brief hypocapnic challenge via controlled hyperventilation, and (6) having signed informed consent by a patient’s next-of-kin. The exclusion criteria were the following: the existence of contraindications for invasive cerebral monitoring, signs of brain death, and lack of signed consent. Measurements have been repeated on the same patient up to three times.

The clinical research ethics committee of the hospital (ACU-AT-203/2012-3531) has reviewed and approved the study protocol, which complied with the Declaration of Helsinki.^56^ The Brain Trauma Foundation clinical guidelines (2016 version) have been followed in patient management.^57^ In cases of patients with refractory intracranial hypertension and no significant volume space-occupying lesions (>25  cc), decompressive craniectomy has been performed.

### 2.2 Optical Device

The hybrid diffuse optical device (TRS, DCS and control modules) described in Refs. 48 and 58 has been used. The TRS module employs pulsed laser light at 687 and 830 nm (PicoQuant, GmbH, Germany) at 3 to 5 mW, that is, collected at an interfiber distance of 3 cm after passing through the tissue by hybrid photomultipliers (HPM-100-50, Becker & Hickl GmbH, Germany) and processed by two TCSPC cards (SPC-130, Becker & Hickl GmbH, Germany). The DCS module uses continuous-wave laser light at 785 nm (iBEAM-SMART, Toptica Photonics AG, Germany) at 28 mW, which is detected at a 2.5 cm distance by four 4-channel single-photon counting modules (SPCM-AQ4C, Excelitas Technologies, USA), providing a total of 16 detection channels (8 per cerebral hemisphere). The sampling rates were 10 Hz and ∼40  Hz, respectively. Both units work simultaneously, with a high-precision synchronization (≪100  milliseconds), and are designed to acquire data on two different locations, i.e., the two frontal lobes, simultaneously. For each location, the source and detector fibers are arranged in a custom-prepared black skin-compatible probe (ethylene-vinyl acetate copolymer, Materials World, ES). A 10 Hz periodic signal provides the joint time-base for the two optical units, which has also been used for synchronization purposes with other standard clinical monitors. Further details can be found in Ref. 59.

### 2.3 Clinical Instrumentation and Data

Standard monitors (either Solar 8000M/i, GE Healthcare, ES or IntelliVue MX800 or MX750, Koninklijke Philips N.V., NL) have been used to obtain arterial blood pressure (ABP), mean arterial blood pressure (MABP), blood oxygen saturation, and heart rate physiological signals.

An intraparenchymal catheter (Camino^®^, Integra NeuroCare, LifeSciences, USA or Neurovent^®^-PTO Raumedic^®^ AG multiparameter sensors, DE) has recorded ICP. The sensor has been placed in the most affected cerebral hemisphere, provided there was no craniectomy. If a craniectomy was present, it has been inserted into the contralateral hemisphere. In cases of diffuse injury without craniectomy, the sensor has been placed in the right cerebral hemisphere.

PowerLab hardware and LabChart software (versions 6 and 8.1, ADInstruments, NZ) have been used to integrate all clinical signals (sampling rate: 400 Hz) with the same timebase, including the synchronization signal provided by the optical device.

Throughout the protocol, arterial blood samples have been extracted from the patients and analyzed by a co-oximeter (GEM Premier 4000, Werfen, ES), providing ${\mathrm{PaCO}}_{2}$ levels.

Additional demographic and clinical data—including sex, age, initial Glasgow Coma Scale (GCS) score, presence of decompressive craniectomy, comorbidities, relevant medical history, surgical evacuation of mass lesions, and cause of TBI—have been extracted from patient records.

### 2.4 Optical Data Acquisition and Hyperventilation Intervention Protocol

After careful examination of the patient’s head computed tomography (CT) scans, the two optical probes have been positioned over the frontal lobes in locations as symmetrical as possible. Areas of skull fractures, subcutaneous edema, or blood pooling have been specifically avoided, as these morphological disruptions can invalidate the assumptions of the light-transport models and confound the optical signals.^49^ Furthermore, probes have not been placed over open wounds or surgical plasters. In cases where tissue disruption near the probe site resulted in excessive light absorption or insufficient signal quality, the sessions have been subsequently excluded based on the objective SNR and count-rate metrics described in Sec. 2.5. The probes have been secured to the scalp using self-adherent elastic bandages, specifically Kinesiology tape (Classic, Kintex, DE) and Coban wrap (Coban™, 3M™, USA). Patients have been kept in a recumbent position with the backrest elevated to approximately 30 deg, a posture recommended for patients with TBI to optimize cerebral perfusion and while reducing ICP.

The protocol has included a baseline period under stable physiological conditions (15 min), a paradigm period with hyperventilation (30 min), and a recovery period (30 min). These durations have been approximate and have been adjusted based on clinical care priorities and staff availability. The HV paradigm has been induced by the clinical staff through the modification of mechanical ventilator settings, specifically by increasing the respiratory rate and/or the tidal volume. The goal has been to induce a hypocapnia with a targeted reduction of ${\mathrm{PaCO}}_{2}$ of 5 to 6 mmHg from baseline while ensuring levels remained above 30 mmHg for safety. The initiation of sufficient HV and hypocapnia has been confirmed via arterial blood gas sample analysis (GEM Premier 4000, Werfen, ES) with samples collected approximately 10 min after the onset of each phase of the protocol. If HV has been insufficient and ${\mathrm{PaCO}}_{2}$ levels could be further reduced within safe limits, the ventilator settings have been modified by the clinical staff, and the blood gas sample analysis has been repeated to confirm the target level. At the end of HV, the clinicians have reinstated the mechanical ventilator settings, targeting the restoration of baseline values. The study protocol, as approved by the ethical committee, has allowed for repeated measurements up to three times during a patient’s hospitalization. However, although some subjects have undergone multiple sessions, the majority have been monitored only once. This has been primarily due to clinical care priorities and patient trajectory. For instance, sessions have been limited by the early removal of invasive monitors, the need for off-ward diagnostic assessments (e.g., CT or MRI scans) or the transition of the patient toward weaning from sedation. To ensure adequate separation between repeated protocols on the same patient when they have occurred, a sufficient time interval (∼1  h between HV periods) has been maintained. Additional details about the protocol are provided in Ref. 48. It must be noted that the purpose of this protocol has been to evaluate the hemodynamic changes induced by HV and has not been therapeutic, as discussed in Ref. 48. Throughout the procedure, events such as patient movements have been annotated both via a custom-built software routine and with handwritten notes to assist in identifying artifacts during data analysis. It is important to emphasize that this paradigm has served no therapeutic purpose and no clinical decisions have been made based on the results. The effect of this paradigm on cerebral hemodynamics of this exact population has been thoroughly described in our previous publication.^48^

### 2.5 Data Analysis

Custom-built scripts have been used for data processing and statistical analyses, implemented partly in MATLAB (Release 2018b, The MathWorks, Inc. Natick, Massachusetts, United States) and partly in RStudio (R v4.0.1, R Core Team, 2019, RStudio Inc., v1.2.5042, USA^60^). The relevant R packages included: *ggplot2* (v. 3.3.0), *stats* (v. 3.6.3), *R.matlab* (v. 3.6.2), *grid* (v. 3.6.3), *gridExtra* (v. 2.3), and *qdapTools* (v. 1.3.3).

Tissue optical properties (reduced scattering and absorption coefficients, $\mu_{s}^{\prime }$ and $\mu_{a}$ respectively) have been derived through a two-step analysis from TRS distributions of time of flight, assuming a semi-infinite homogeneous medium. Data have been fitted to the model as described in Refs. 59 and 61–63 and therein. Oxy- and deoxy-hemoglobin concentrations have then been calculated using a linear system based on the absorption coefficients and molecular extinction coefficients at 687 and 830 nm to obtain tissue oxygen saturation (${\mathrm{StO}}_{2}$).^64^ DCS data have been fitted to the semi-infinite solution of the correlation diffusion equation according to the procedure in Refs. 64 and 65, using the TRS-based optical properties as input to the correlation diffusion equation to compute the blood flow index (BFI), a CBF surrogate.^64^^,^^66^ As the DCS laser operates at a different wavelength (785 nm) than the TRS sources (687 and 830 nm) to prevent cross-talk, the $\mu_{s}^{\prime }$ and $\mu_{a}$ input values required for BFI extraction have been estimated via linear interpolation (averaging) of the values obtained from the two TRS channels.^67^^–^^69^ Further details about the data processing can be found in Ref. 59 and in the Appendix Sec. 6.2.

For subsequent analysis, optical data (i.e., a time-trace) from the two cerebral hemispheres have been treated as independent. Consequently, all derived indices have been calculated and assessed separately for each hemisphere. Data rejection criteria based on strict quality control have been detailed in Ref. 48 and applied. Artifact removal procedures—also described therein—have addressed disruptions primarily due to clinical procedures and patient movement. Briefly, artifacts have been identified through event markers recorded during measurements and via visual inspection of anomalous time-trace patterns during post-processing. Affected data points have been excluded from analysis and replaced with “not available” (NA) values.

For each measurement, the core variables assessed have been ICP, MABP, ${\mathrm{StO}}_{2}$, and BFI. These have been temporally aligned using the shared time-base signal, then re-binned into nonoverlapping 5-s windows, within which all samples, originally acquired at different sampling rates, have been averaged to obtain time series with a uniform sampling frequency. CPP has been subsequently computed using Eq. (1). A summary of all measured variables is presented in Table 1.

**Table 1:** Summary of the measured parameters and their acronyms.

Acronym	Meaning	Acquisition method
**BFI**	Blood flow index	DCS
${\mathrm{StO}}_{2}$	Tissue oxygen saturation	TRS
**MABP**	Mean arterial blood pressure	Invasive arterial line
**CPP**	Cerebral perfusion pressure	Derived from ICP and MABP
**ICP**	Intracranial pressure	Invasive intraparenchymal catheter
${\mathrm{PaCO}}_{2}$	Arterial pressure of carbon dioxide	Blood gas sample analyzer

Clinical and demographic data have been summarized as follows: numerical variables are reported as median (interquartile range, between the 25th and 75th percentiles) (IQR), whereas categorical variables are reported as as counts of cases and percentages (n [%]).

#### 2.5.1 Calculation of cerebral autoregulation indices

To prove our first hypothesis, seven distinct CAR indices have been computed based on established methods in the literature.^16^^,^^27^^,^^70^ Table 2 summarizes the parameters and their corresponding acronyms. Note that six indices have been calculated separately for each cerebral hemisphere due to the presence of bilateral optical probes, resulting in a total of 13 CAR indices being evaluated.

**Table 2:** Summary of calculated CAR indices and their acronyms. BFI, blood flow index; ${\mathrm{StO}}_{2}$, tissue oxygen saturation; ICP, intracranial pressure; MABP, mean arterial blood pressure; CPP, cerebral perfusion pressure. *For ${\mathrm{DCSx}}_{\mathrm{CPP}}$, no references demonstrating a continuous sliding-window calculation could be found, but correlation plots (as in Lassen’s method) for static CAR can be found in Ref. 71. Moreover, there are publications based on CBF by TCD.^21^^,^^72^ **Only publications based on TCD could be found, computing the Mx (between CPP and middle cerebral artery velocity—CBFV—measured via TCD) or FIx index (computed from ICP and CBFV) instead.^73^^,^^74^ ***No references could be found.

Acronym	Meaning	References
PRx	Pressure reactivity index	6, 19, 20, 27
${\;\mathrm{DCSx}}_{\mathrm{MABPleft}}$	Diffuse correlation spectroscopy-based CAR index calculated from BFI and MABP for the left cerebral hemisphere	42, 70, 71, 75
${\mathrm{DCSx}}_{\mathrm{MABPright}}$	Diffuse correlation spectroscopy-based CAR index calculated from BFI and MABP for the right cerebral hemisphere	
${\mathrm{DCSx}}_{\mathrm{CPPleft}}$	Diffuse correlation spectroscopy-based CAR index calculated from BFI and CPP for the left cerebral hemisphere	*
${\mathrm{DCSx}}_{\mathrm{CPPright}}$	Diffuse correlation spectroscopy-based CAR index calculated from BFI and CPP for the right cerebral hemisphere	
${\mathrm{DCSx}}_{\mathrm{ICPleft}}$	Diffuse correlation spectroscopy-based CAR index calculated from BFI and ICP for the left cerebral hemisphere	**
${\mathrm{DCSx}}_{\mathrm{ICPright}}$	Diffuse correlation spectroscopy-based CAR index calculated from BFI and ICP for the right cerebral hemisphere	
${\mathrm{COx}}_{\mathrm{MABPleft}}$	Cerebral oximetry CAR index calculated from ${\mathrm{StO}}_{2}$ and MABP for the left cerebral hemisphere	29–31, 33, 42, 76, 77
${\mathrm{COx}}_{\mathrm{MABPright}}$	Cerebral oximetry CAR index calculated from ${\mathrm{StO}}_{2}$ and MABP for the right cerebral hemisphere	
${\mathrm{COx}}_{\mathrm{CPPleft}}$	Cerebral oximetry CAR index calculated from ${\mathrm{StO}}_{2}$ and CPP for the left cerebral hemisphere	6, 33, 78
${\mathrm{COx}}_{\text{CPPright}}$	Cerebral oximetry CAR index calculated from ${\mathrm{StO}}_{2}$ and CPP for the right cerebral hemisphere	
${\mathrm{COx}}_{\text{ICPleft}}$	Cerebral oximetry CAR index calculated from ${\mathrm{StO}}_{2}$ and ICP for the left cerebral hemisphere	***
${\mathrm{COx}}_{\text{ICPright}}$	Cerebral oximetry CAR index calculated from ${\mathrm{StO}}_{2}$ and ICP for the right cerebral hemisphere	

First, we have calculated the PRx, a widely used invasive measure derived from ICP and MABP.^19^ We have then included the following indices: ${\mathrm{DCSx}}_{\mathrm{CPP}}$ as correlation between BFI and CPP, ${\mathrm{DCSx}}_{\mathrm{ICP}}$ as correlation between BFI and ICP, ${\mathrm{DCSx}}_{\mathrm{MABP}}$ as correlation between BFI and MABP, ${\mathrm{COx}}_{\mathrm{CPP}}$ as correlation between ${\mathrm{StO}}_{2}$ and CPP, ${\mathrm{COx}}_{\mathrm{ICP}}$ as correlation between ${\mathrm{StO}}_{2}$ and ICP, and ${\mathrm{COx}}_{\mathrm{MABP}}$ as correlation between ${\mathrm{StO}}_{2}$ and MABP. To our knowledge, no previous studies have computed COx or DCSx using ICP likely due to its invasive nature and limited availability. However, given its prior use in TCD-based blood flow studies and its methodological analogy with our approach,^73^^,^^74^ we have considered its inclusion both feasible and informative. For example, these indices could enable one to explore vasogenic behavior intrinsic to the cerebral circulation, a method particularly relevant for identifying pathology in patients with normal mean ICP values, a critical diagnostic gap.

All indices have been calculated using a moving Pearson’s correlation coefficient between the two relevant signals,^79^ applying a sliding window of 300 s with a step size of 5 s across the full measurement duration. The 300-s window has been selected based on prior evidence, suggesting it is commonly sufficient to assess CAR at rest.^80^ A 5-s step has ensured smooth, continuous time series with high overlap between consecutive windows. Calculations have been performed in RStudio using the R package *zoo* (v. 1.8-7), with correlations computed via the *rollapply* function. The resulting time vector begins at 300 s, corresponding to the end of the first full window. Of note, this correlation-based sliding-window approach is conceptually aligned with recent CARNet-related methodology,^4^ although a finer step size (5 s rather than 10 s) has been used here.

For all DCSx and COx-based indices, calculations have been performed separately for each cerebral hemisphere, resulting in hemisphere-specific indices (e.g., ${\mathrm{DCSx}}_{\mathrm{MABPleft}}$ and ${\mathrm{DCSx}}_{\mathrm{MABPright}}$; ${\mathrm{COx}}_{\mathrm{MABPleft}}$ and ${\mathrm{COx}}_{\mathrm{MABPright}}$). PRx, by contrast, has been evaluated only for the hemisphere in which the invasive ICP probe was placed. All indices have been plotted over time to examine their temporal dynamics.

### 2.6 Statistical Analysis

Initially, we have compared the CAR indices before HV and after its end, specifically by examining values obtained during baseline and recovery periods. For each measurement, two 5-min intervals have been selected from each time-trace: one during the baseline phase and one during recovery, using the same intervals for all variables within each session. This 5-minute duration has been shown to be sufficient for reliably assessing CAR.^3^ The first interval has been extracted from the baseline phase, defined as the 5 min ending 150 s before the event mark indicating the start of HV. The second interval has been selected starting 150 s after the end of the HV paradigm and continuing for 5 min. The 150-s buffer around protocol transitions has been applied to reduce the influence of transient physiological changes on the 300-s moving correlation window. If artifacts were present within the defined intervals, the segments have been slightly shifted in time to ensure that the full duration has been preserved without including corrupted data. For each interval, the median values of the CAR indices have been computed. These pre- and post-HV intervals have been selected to ensure methodological consistency and physiological interpretability, being stable phases of the protocol and minimizing confounding effects associated with active HV. This approach aligns with common clinical/experimental practice, in which CAR is inferred from steady-state conditions rather than during transient perturbations. For clarity, references to HV-related effects throughout the paper denote comparisons between baseline and recovery periods, unless explicitly stated otherwise.

This procedure has been repeated across all measurements and indices, resulting in a comprehensive dataset. To visualize the distribution of these data points across the group, we have used semi-violin plots and boxplots (including median, 25th and 75th percentiles). To address our second hypothesis, we have then tested for statistical significance of changes in each CAR index between the two time periods using linear mixed-effects models for repeated measurements. The significance threshold has been set at a type I error of 0.05. This has been implemented in RStudio using the R package *lme4* (v. 1.1-31). In addition to significance testing, we have reported the median and interquartile range (IQR) for each index and physiological variable in both baseline and recovery periods.

To address our third hypothesis, to assess whether DO-based indices exhibit lower variability, and thus potentially greater reliability, than PRx at a group level, we have calculated the standard deviation and IQR for each CAR index within the two time windows. These measures of uncertainty have been visualized via boxplots, separated by baseline and recovery periods. DO-based indices with lower median variability and narrower percentile range compared with PRx have been considered to show lower uncertainty. Wilcoxon signed-rank tests for paired samples have been used to assess whether the median value of each CAR index was significantly lower than the median of the PRx distribution (alternative = “less” in the wilcox.test function). Each CAR index has been tested individually. A p-value (p) of less than 0.05 has been considered statistically significant. Significant changes in ${\mathrm{PaCO}}_{2}$, ICP, and CPP between pre- and post-HV values have been assessed using Wilcoxon signed-rank tests for paired samples, with a significance threshold of p<0.05.

In addition to group-level analysis, we have also performed subject-specific assessments. For each measurement, we have evaluated whether a significant change (increase or decrease) has occurred relative to the corresponding baseline. This has been done using the Wilcoxon signed-rank test, comparing the sample distribution from the recovery interval against the median baseline value. Significance has been defined as a p-value less than 0.05. This analysis has been implemented using the *wilcox.test* function in R. For every variable, the observed trend has been categorized as either an increase (↑), decrease (↓), no change (–), or not available (NA).

To assess the consistency of CAR responses, based on the subject-specific assessment, we have calculated an “agreement score” for each measurement, defined as the percentage of indices exhibiting a statistically significant change in the same direction. This score reflected inter-measurement concordance and served as an indicator of CAR assessment reliability. With 13 CAR indices (seven distinct features, six bilaterally) being evaluated, complete agreement corresponds to 100%. The “agreement score” has been computed as the proportion of nonmissing variables sharing the most frequent directional trend (increase, decrease, or no change), all treated as equally meaningful. In cases of ties, one tied trend has been selected to provide a conservative estimate. Formally:


$$\text{Agreement score}=\frac{\text{Number of variables}\;\text{with}\;\text{majority trend}}{\text{Total number of non}\text{-}\mathrm{NA}\;\text{variables}}\times 100\%.$$

This metric offers a unified measure of directional agreement across multimodal CAR indices. The results have been categorized as high agreement for scores equal to or above 75%, medium agreement for scores between 50% and 75%, and mild agreement for scores below 50%.

To evaluate the architectural independence of the pooled consensus metric and mitigate the risk of numerical dominance by clustered sensor types, secondary family-wise and hemispheric sub-group evaluations have been executed. The 13 metrics have been isolated into regional clusters based on their underlying physiological signal modality, microvascular BFI (DCSx family, n=6), and ${\mathrm{StO}}_{2}$ (COx family, n=6) and separated by anatomical monitoring site (left versus right cerebral hemisphere, which we have called “optical agreement scores”). For each specific subgroup session, a local consensus direction (↑, ↓, or −) has been established based on the majority frequency of its nonmissing component indices. In instances of an exact directional tie, a functional decrease (↓) has been selected as the default fallback parameter. The sub-group agreement score has been subsequently computed as the percentage of available measurements matching this local dominant trend. This provided six additional agreement scores (AS) with their own direction (i.e., ${\mathrm{DCSx}}_{\mathrm{LAS}}$, ${\mathrm{DSCx}}_{\mathrm{dir}}$). Furthermore, cross-modality alignment has been assessed by directly tracking the percentage of nonmissing optical indices that perfectly mirrored the directional vector of the global, single-probe pressure reactivity index (PRx).

## 3 Results

### 3.1 Patient Characteristics

Out of the 33 TBI patients that were recruited for the original study,^48^ we retrospectively selected seventeen who had undergone continuous MABP monitoring with arterial lines. This has resulted in a total of twenty measurement sessions acquired between November 2017 and May 2021. Specifically, 15 patients have undergone a single measurement session, one patient has completed two sessions, and one patient three sessions. One of these patients did not undergo invasive ICP monitoring. As optical data have been collected from both cerebral hemispheres, this yielded 20 measurements per hemisphere. However, one DCS acquisition from the right hemisphere and four from the left hemisphere have been excluded due to poor signal quality, defined as a detected photon count-rate below 10 kHz (see Sec. 2.5 and references for details). For TRS data, two acquisitions from the right hemisphere and six from the left hemisphere have been discarded due to insufficient signal-to-noise ratio (SNR), as defined in Ref. 48.

Table 3 summarizes the clinical and demographic characteristics of the selected patients, reporting the number (n) and percentage for each variable out of the total sample size (n=17). The median age of the group was 32 years (min: 16, max: 61 years). Thirteen patients were male, and four were female. We note that for the majority of patients (15/17) and measurements (18/20), the invasive ICP sensor has been placed in the right hemisphere. Consequently, right-sided optical measures are primarily representative of the ipsilateral side relative to the global ICP-based gold standard (PRx), whereas in this study, optical CAR indices are categorized by hemisphere (left versus right). We note that in this type of injury, there is no well-defined hemispheric effect.

**Table 3:** Demographic and clinical information of the seventeen included patients. n indicates the number of subjects presenting a certain characteristic, whereas the value in brackets corresponds to the percentage. *GCS for one subject was missing.

		n [%]
**Sex**	Female	4 (23.5)
	Male	13 (76.5)
**Age [median years(IQR)]**	32 (23,47)	
**GCS [median(IQR)]**	4 (3,9)*	
**Causes of TBI**	Traffic accident	8 (48)
	Pedestrian	2 (12)
	Fall from same-level	3 (17)
	Fall from height>1 m	3 (17)
	Other	1 (6)
**Decompressive craniectomy**	Left hemisphere	1 (6)
	None	16 (94)
**Mass lesions**	Evacuated	11 (65)
	Nonevacuated	6 (35)
**Medical history**	Chronic obstructive pulmonary disease	1 (6)
**Comorbidities**	Enolism	6 (35)
	Dyslipidemia	1 (6)
	Depression	1 (6)
	Hypertension	3 (17)
	Mild factor X coagulation deficiency	1 (6)

### 3.2 Representative Time-Traces of Clinical Data and Derived CAR Indices

**Fig. 1:**
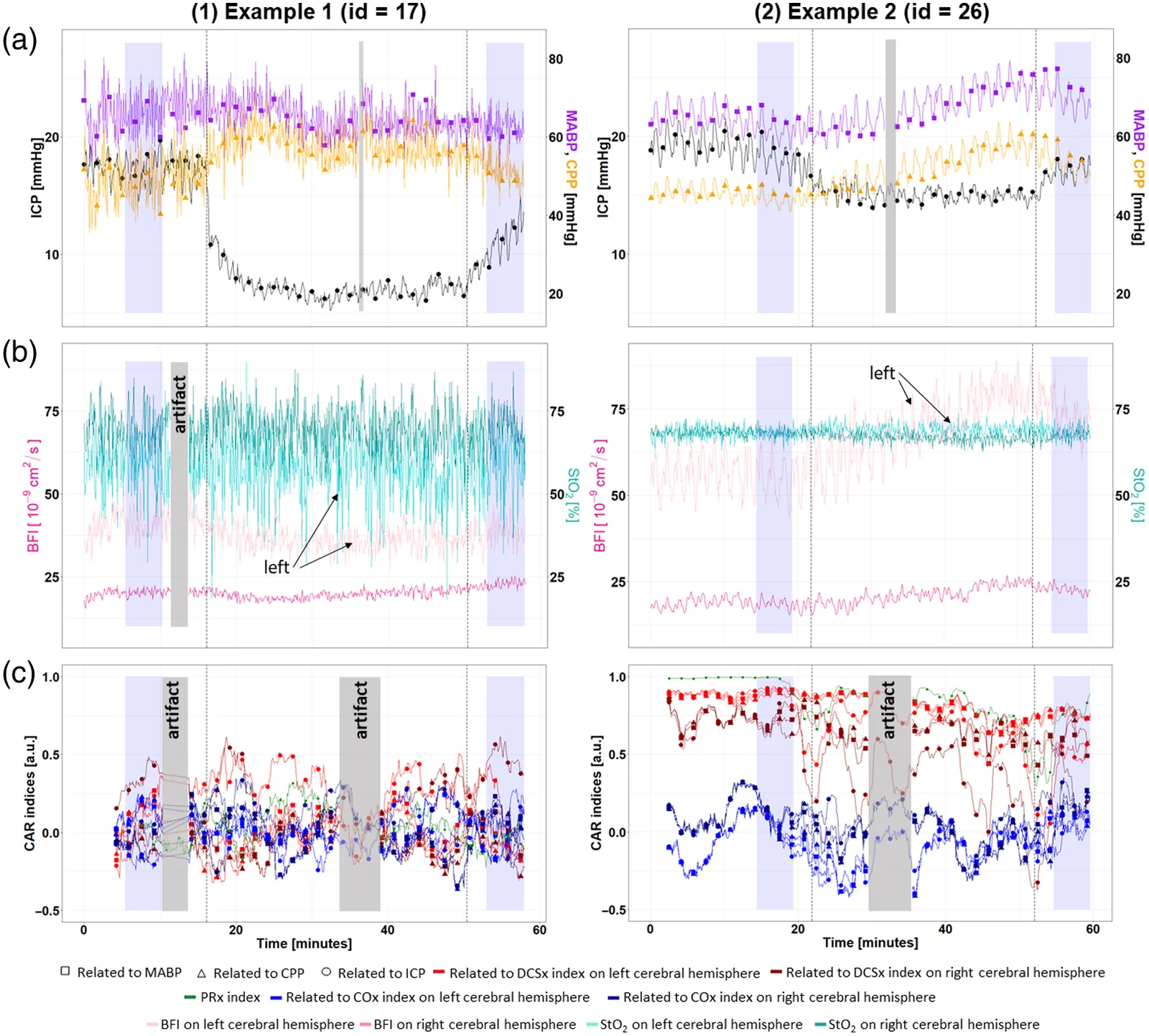
Representative physiological and cerebral autoregulation time-series data (ICP, CPP, MABP, BFI, ${\mathrm{StO}}_{2}\;{\mathrm{DCSx}}_{\mathrm{CPP}}$, ${\mathrm{DCSx}}_{\mathrm{MABP}}$, ${\mathrm{DCSx}}_{\mathrm{ICP}}$, ${\mathrm{COx}}_{\mathrm{ICP}}$, ${\mathrm{COx}}_{\mathrm{MABP}}$, ${\mathrm{COx}}_{\mathrm{CPP}}$, PRx). Two clinical cases are presented for comparison: Example 1 (column 1) and Example 2 (column 2). Vertical dashed lines delineate the transition between baseline (HV beginning) and recovery (HV end) periods. Light-blue shaded areas indicate the 5-min segments selected for steady-state analysis (calculation of average values prior and after the end of the HV paradigm), whereas gray areas denote identified artifacts. Some parameters are differentiated per cerebral hemisphere, left or right with light and dark shades, respectively. Row (a): recordings of MABP (purple), CPP (orange), and ICP (black). Row (b): traces for BFI (shades of pink) and ${\mathrm{StO}}_{2}$ (shades of teal). Arrows indicate measurements obtained from the left cerebral hemisphere; consistent with the legend, darker tones represent the right hemisphere. Row (c): CAR indices including PRx (green), COx-related (blue shades), and DCSx-related (red shades). Consistent with rows (a) and (b), left-sided indices are represented by lighter lines, whereas right-sided indices use darker lines. Individual traces are further shown in Fig. 6 for closer inspection. HV, hyperventilation; MABP, mean arterial blood pressure; CPP, cerebral perfusion pressure; ICP, intracranial pressure; PRx, pressure reactivity index; DCSx, cerebral autoregulation index calculated from BFI; COx, cerebral autoregulation index calculated from ${\mathrm{StO}}_{2}$; BFI, blood flow index; ${\mathrm{StO}}_{2}$, tissue oxygen saturation.

Figure 1 presents representative time-traces acquired during two independent measurement sessions from two different subjects. In “Example 1,” data are shown from a 23-year-old male TBI patient with bilateral cerebral lesions and an initial GCS score of 3. In “Example 2,” the patient is a 16-year-old female with a lesion localized to the left cerebral hemisphere and an initial GCS of 7. Notably, the latter also displays low-frequency periodic oscillations in most of the recorded signals.

Following the initiation of the HV paradigm, ICP has decreased in both cases (top row, black traces), in line with established physiological responses reported in the literature. MABP and CPP have shown minimal variations over time. There are distinct behaviors observable in the CAR indices (bottom row). For instance, “Example 1” displays a relatively stable pattern oscillating around a mean value, and all indices are similar. By contrast, “Example 2” shows two distinct values where PRx and DCSx-related indices show higher values, possibly impaired CAR, whereas COx-related indices show lower values. Furthermore, there is a directional trend toward lower values in a subset of indices toward the end of the HV period, although it is not consistent in amplitude across all indices.

To better illustrate how the indices vary depending on whether they are computed from MABP, CPP, or ICP, additional visualizations of these examples are provided in the Appendix Sec. 6.1 (Fig. 6).

### 3.3 Group Results

At the group level, ${\mathrm{PaCO}}_{2}$ has significantly decreased from a baseline median of 38 (35, 40) mmHg to 35 (33, 38) mmHg (p<0.001) after the end of the HV period, reflecting a median reduction of at least ∼2 mmHg. ICP has shown a significant decrease from 7.8 (3.5, 15) mmHg to 6.2 (2.9, 14) mmHg (p<0.01), whereas MABP has significantly increased from 72 (66, 85) mmHg to 77 (70, 91) mmHg. CPP has risen from 69 (51, 74) mmHg to 71 (63, 82) mmHg (p<0.01). The total duration of the measurement sessions has been 88±17  min (range: 70 to 138 min). The overall HV intervention has lasted on average 40±15  min, including periods of respiration settings readjustment, while it has achieved sufficient depth for 30±2  min.

**Fig. 2:**
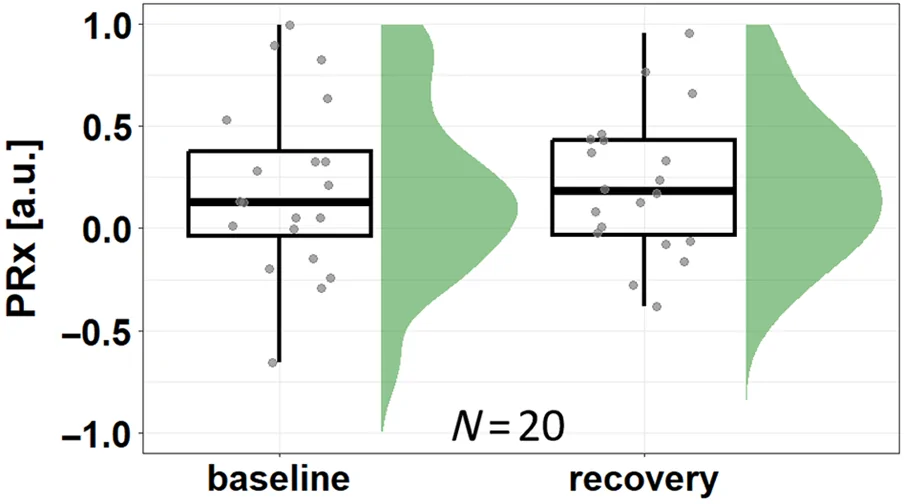
Distributions of the PRx index during the state of baseline, prior to HV paradigm, and during the state of recovery, after HV. HV: hyperventilation. PRx: pressure reactivity index. N: number of samples per each distribution (same N for baseline and recovery).

**Fig. 3:**
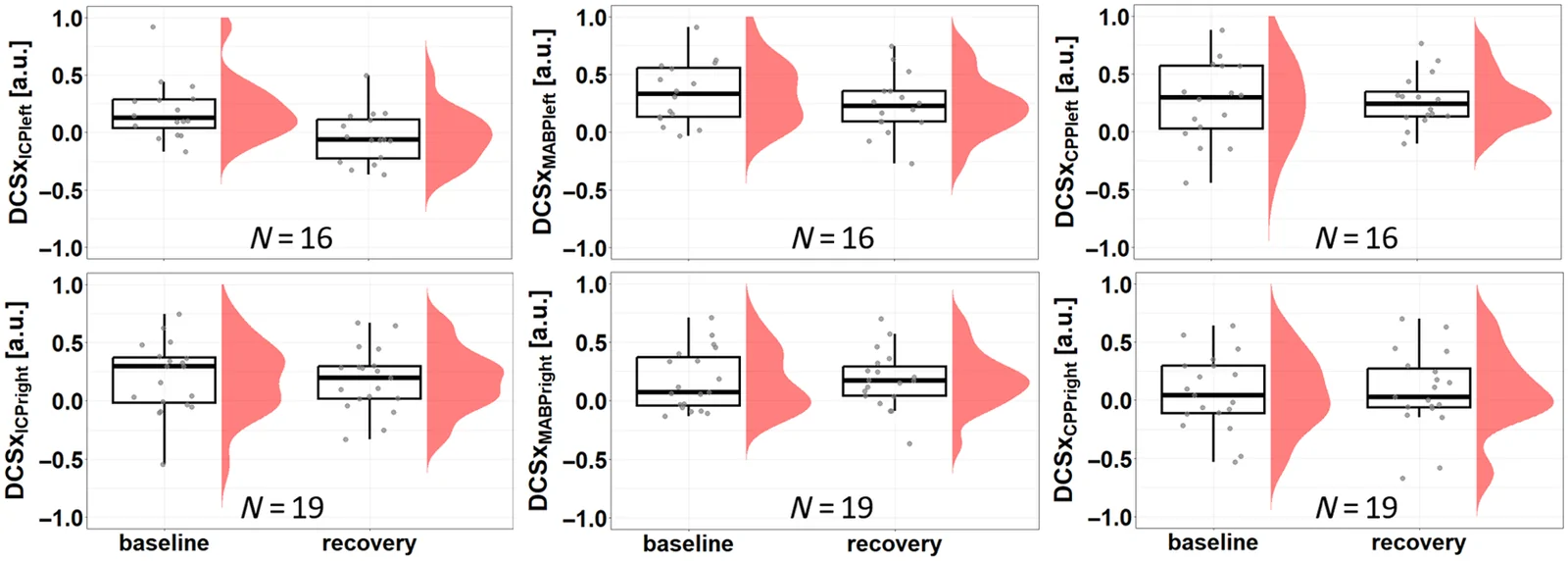
Distributions of the DCSx CAR-related indices during the state of baseline, prior to HV paradigm, and during the state of recovery, after HV, differentiated between cerebral hemisphere (left or right): ${\mathrm{DCSx}}_{\mathrm{CPP}}$, ${\mathrm{DCSx}}_{\mathrm{MABP}}$, ${\mathrm{DCSx}}_{\mathrm{ICP}}$. HV, hyperventilation; ICP, intracranial pressure; MABP, mean arterial blood pressure; ${\mathrm{DCSx}}_{\mathrm{CPP}}$, cerebral autoregulation index calculated from BFI and CPP; ${\mathrm{DCSx}}_{\mathrm{MABP}}$, cerebral autoregulation index calculated from BFI and MABP; CPP, cerebral perfusion pressure; BFI, blood flow index; N, number of samples per each distribution (same N for baseline and recovery).

**Fig. 4:**
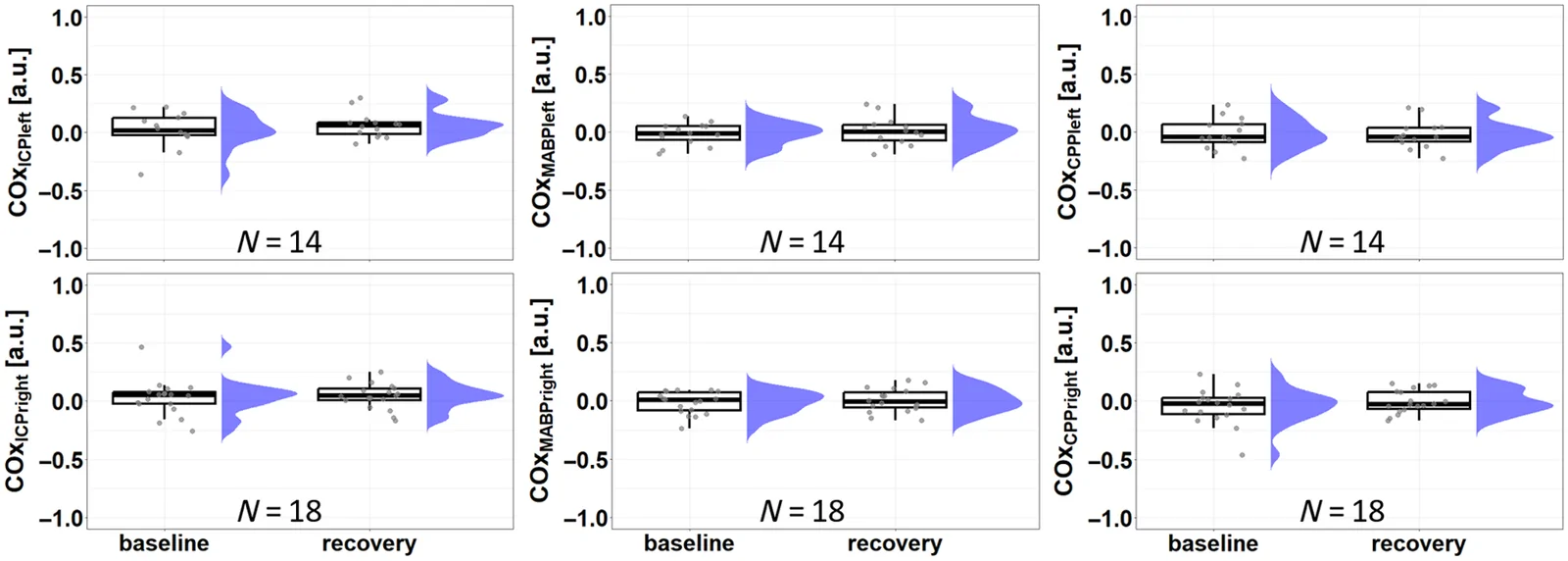
Distributions of the COx CAR-related indices (${\mathrm{COx}}_{\mathrm{MABP}}$, ${\mathrm{COx}}_{\mathrm{CPP}}$, and ${\mathrm{COx}}_{\mathrm{ICP}}$) during the state of baseline, prior to HV paradigm, and during the state of recovery, after HV. Indices are separated per cerebral hemisphere: left or right. HV, hyperventilation; ICP, intracranial pressure; MABP, mean arterial blood pressure; CPP, cerebral perfusion pressure; ${\mathrm{COx}}_{\mathrm{MABP}}$, cerebral autoregulation index calculated from ${\mathrm{StO}}_{2}$ and MABP; ${\mathrm{COx}}_{\mathrm{ICP}}$, cerebral autoregulation index calculated from ${\mathrm{StO}}_{2}$ and ICP; ${\mathrm{COx}}_{\mathrm{CPP}}$, cerebral autoregulation index calculated from ${\mathrm{StO}}_{2}$ and CPP; N, number of samples per each distribution (same N for baseline and recovery).

Figures 2, 3 and 4 illustrate the distributions of CAR indices during baseline and recovery windows. The values are constrained within the theoretical bounds of −1 to 1.

The DCS-derived indices have shown heterogeneous responses between hemispheres and systemic drivers. Notably, ${\mathrm{DCSx}}_{\mathrm{ICP}}$ has exhibited a reduction in both hemispheres during recovery, with a shift from 0.13 to -0.06 on the left side. Conversely, COx indices have remained remarkably stable across all subjects and conditions, demonstrating limited variability and near-zero median values throughout the study. Detailed medians and interquartile ranges for all 13 indices are provided in Table 4.

**Table 4:** Summary of median and IQR for the distributions of cerebral autoregulation indices during baseline and recovery periods. IQR, interquartile range.

Index	Baseline [median (IQR)]	Recovery [median (IQR)]
${\;\mathrm{DCSx}}_{\mathrm{MABPleft}}$	0.33 (0.13, 0.56)	0.22 (0.09, 0.35)
${\mathrm{DCSx}}_{\mathrm{MABPright}}$	0.07 (−0.04, 0.37)	0.17 (0.04, 0.29)
${\mathrm{DCSx}}_{\mathrm{CPPleft}}$	0.30 (0.03, 0.57)	0.24 (0.13, 0.35)
${\mathrm{DCSx}}_{\mathrm{CPPright}}$	0.04 (−0.11, 0.30)	0.03 (−0.06, 0.27)
${\mathrm{DCSx}}_{\mathrm{ICPleft}}$	0.13 (0.04, 0.29)	−0.06 (−0.23, 0.11)
${\mathrm{DCSx}}_{\mathrm{ICPright}}$	0.30 (−0.02, 0.37)	0.19 (0.02, 0.29)
${\mathrm{COx}}_{\mathrm{MABPleft}}$	−0.01 (−0.07, 0.05)	0.00 (−0.07, 0.06)
${\mathrm{COx}}_{\mathrm{MABPright}}$	0.01 (−0.08, 0.07)	0.00 (−0.06, 0.07)
${\mathrm{COx}}_{\mathrm{CPPleft}}$	−0.04 (−0.09, 0.07)	−0.04 (−0.08, 0.04)
${\mathrm{COx}}_{\mathrm{CPPright}}$	−0.03 (−0.10, 0.03)	−0.03 (−0.07, 0.08)
${\mathrm{COx}}_{\mathrm{ICPleft}}$	0.02 (−0.02, 0.12)	0.06 (−0.01, 0.09)
${\mathrm{COx}}_{\mathrm{ICPright}}$	0.05 (−0.02, 0.08)	0.04 (0.01, 0.10)
PRx	0.13 (−0.04, 0.38)	0.18 (−0.03, 0.38)

Although the full set of 20 measurement sessions has been utilized for distribution visualizations, single-subject analyses, and “agreement scores” (unless data were unavailable for a specific parameter), the primary statistical analysis has required a more selective approach. In fact, due to a minimal random effect and the presence of only two subjects with repeated measurements, we have encountered a "singular fit" in our original mixed-effects models, rendering them statistically unreliable. As a result, we have excluded the repeated sessions and retained only the first available measurement per subject (n=17) to proceed with paired Wilcoxon signed-rank tests. This choice has ensured a homogeneous group where every data point represents a first measurement. To account for multiple comparisons across the 13 CAR indices, p-values have later been adjusted using the Benjamini–Hochberg false discovery rate (FDR) procedure. Adjusted p-values ($p_{\text{adjusted}}$) <0.05 have been considered statistically significant.

The analysis has revealed that while two indices exhibited nominal significance in their raw p-values ($p_{\text{raw}}$), only ${\mathrm{DCSx}}_{\mathrm{ICPleft}}$ has remained statistically significant after multiple comparison adjustment ($p_{\text{raw}}=0.0023$, $p_{\text{adjusted}}=0.030$). Notable trends toward a decrease during recovery have been observed for ${\mathrm{DCSx}}_{\mathrm{MABPleft}}$ ($p_{\text{raw}}=0.0199$, $p_{\text{adjusted}}=0.129$) and ${\mathrm{DCSx}}_{\mathrm{CPPright}}$ ($p_{\text{raw}}=0.151$, $p_{\text{adjusted}}=0.656$), though these have not survived the FDR correction. No significant changes or strong trends have been observed for PRx ($p_{\text{adjusted}}=0.812$), any of the COx-derived indices ($p_{\text{adjusted}}>0.812$ in all cases) or the remaining DCSx parameters, with p>0.05 in all cases. These group-level results are summarized in Table 5.

**Table 5:** Statistical comparison between baseline and recovery periods. $p_{\mathrm{raw}}$ indicates the unadjusted p-values from the paired Wilcoxon signed-rank tests (n=17), whereas $p_{\text{adjusted}}$ represents p-values adjusted for multiple comparisons using the Benjamini–Hochberg false discovery rate (FDR) procedure. * indicates p<0.05.

CAR index	$p_{\text{raw}}$	$p_{\text{adjusted}}$
${\;\mathrm{DCSx}}_{\mathrm{MABPleft}}$	0.0199	0.1293
${\mathrm{DCSx}}_{\mathrm{MABPright}}$	0.3394	0.8116
${\mathrm{DCSx}}_{\mathrm{CPPleft}}$	0.3177	0.8116
${\mathrm{DCSx}}_{\mathrm{CPPright}}$	0.1514	0.6561
${\mathrm{DCSx}}_{\mathrm{ICPleft}}$	0.0023	0.0302*
${\mathrm{DCSx}}_{\mathrm{ICPright}}$	0.5110	0.8116
${\mathrm{COx}}_{\mathrm{MABPleft}}$	0.7407	0.8116
${\mathrm{COx}}_{\mathrm{MABPright}}$	0.7492	0.8116
${\mathrm{COx}}_{\mathrm{CPPleft}}$	0.6333	0.8116
${\mathrm{COx}}_{\mathrm{CPPright}}$	0.9031	0.9031
${\mathrm{COx}}_{\mathrm{ICPleft}}$	0.6890	0.8116
${\mathrm{COx}}_{\mathrm{ICPright}}$	0.6651	0.8116
PRx	0.4301	0.8116

#### 3.3.1 Variability of CAR indices over time

**Fig. 5:**
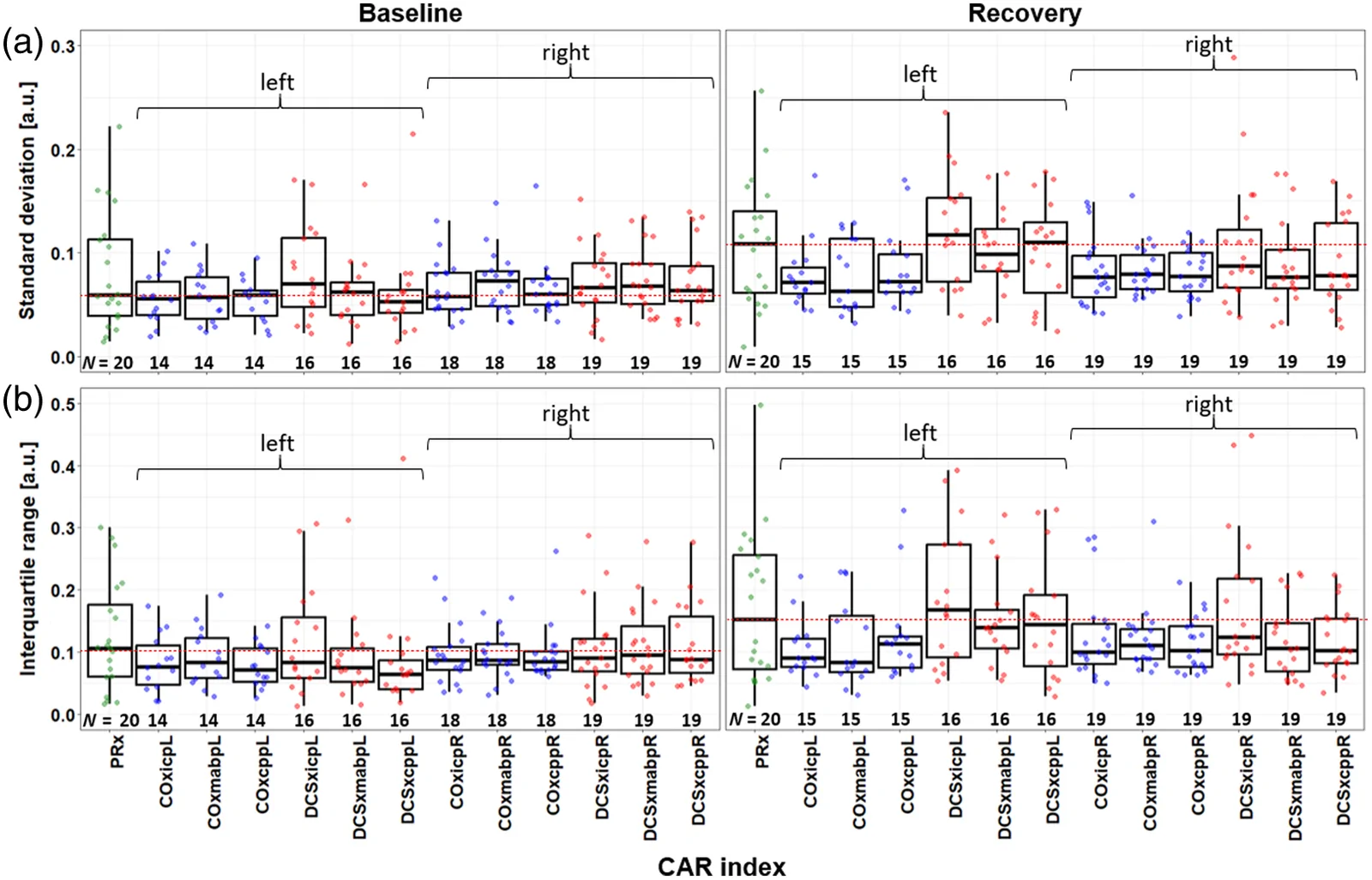
Distributions of the uncertainty quantified as standard deviation (a) and interquartile range (b) for all CAR indices during the state of baseline (left column), prior to HV paradigm, and during the state of recovery (right column), after HV, for PRx (green), DCSx-derived (red) and COx-derived (blue) indices. Boxplot center lines indicate the median, whereas whiskers extend to 1.5 times the interquartile range. Indices are organized by hemisphere, with left-sided parameters grouped first (just after PRx), followed by right-sided parameters beneath overarching brackets, to facilitate regional comparison. Individual data points represent separate measurement sessions, with the total number of valid samples (N) indicated above the x-axis for each distribution. The horizontal red dashed line indicates the median variability of the global PRx index within each state, providing a baseline for comparing the variability across indices.

Figure 5 displays the distributions of variability for each CAR index that has been quantified by the standard deviation and IQR during both baseline and recovery periods (see Sec. 2). Lower values indicate reduced overall uncertainty in the variable, whereas narrower boxplots suggest lower variability of the underlying biosignal itself over the population. To assess whether DO-based variables exhibit greater reliability compared with PRx—which has previously been criticized for occasionally producing unreliable, spurious, and nonphysiological values,^7^ the median of the PRx distributions has been marked with a red dashed line as a threshold. We start with a qualitative analysis based on the observations from this figure. Indices with median values below this line are considered to have lower variability than PRx. Qualitatively, the median values of the distribution for the other indices mostly follow our hypothesis only in the cases of the standard deviation during the recovery period and of the IQR both during baseline and recovery. However, the overall spread of these indices appears comparable to PRx.

This has been further confirmed by statistical analysis of standard deviation distribution revealing that during baseline, ${\mathrm{COx}}_{\mathrm{CPPleft}}$, ${\mathrm{COx}}_{\mathrm{ICPleft}}$, ${\mathrm{COx}}_{\mathrm{MABPleft}}$, and ${\mathrm{DCSx}}_{\mathrm{CPPleft}}$ exhibited significantly lower variability compared with PRx (all p<0.05), whereas other parameters have shown no significant differences (p>0.05). During recovery, only ${\mathrm{COx}}_{\mathrm{ICPleft}}$ and ${\mathrm{COx}}_{\mathrm{MABPleft}}$ have maintained significantly lower variability than PRx (both p<0.05).

When variability has been assessed via IQR, several CAR indices have demonstrated significantly lower values than PRx at baseline: ${\mathrm{COx}}_{\mathrm{CPPleft}}$ (p<0.01), ${\mathrm{COx}}_{\mathrm{ICPleft}}$ (p<0.01), ${\mathrm{DCSx}}_{\mathrm{CPPleft}}$ (p<0.05), and ${\mathrm{DCSx}}_{\mathrm{MABPleft}}$ (p<0.05). Conversely, ${\mathrm{COx}}_{\mathrm{CPPright}}$, ${\mathrm{COx}}_{\mathrm{ICPright}}$, ${\mathrm{COx}}_{\mathrm{MABPleft}}$, and right-sided DCS-derived indices have shown no significant differences (p>0.05). During recovery, a broader pattern of significant reductions in IQR has emerged: ${\mathrm{COx}}_{\mathrm{CPPleft}}$ (p<0.05), ${\mathrm{COx}}_{\mathrm{CPPright}}$ (p<0.05), ${\mathrm{COx}}_{\mathrm{ICPleft}}$ (p<0.05), and ${\mathrm{COx}}_{\mathrm{MABPleft}}$ (p<0.01) all demonstrated significantly lower variability than PRx.

### 3.4 Comparison of the Results on a Patient-by-Patient Basis

As hypothesized, the group response to HV is quite heterogeneous, and we have sought to analyze the results on a patient-by-patient basis. To do so, we have tested the significance of the changes in CAR indices for each patient. The results, summarized as the number of measurements where a significant change has been observed per variable of interest, are presented in Table 6. The results show that different combinations of variables lead to a different number of patients showing changes and, even, to different directions of changes.

**Table 6:** Summary of single-subject significance analysis (Wilcoxon signed-rank test). N represents the number of valid measurements per index. This table quantifies the heterogeneity of individual responses to HV across different modalities.

	Sig. increase	Sig. decrease	No change	N
PRx	8	12	0	20
${\;\mathrm{DCSx}}_{\mathrm{MABPleft}}$	4	11	1	16
${\mathrm{DCSx}}_{\mathrm{MABPright}}$	10	7	2	19
${\mathrm{DCSx}}_{\mathrm{CPPleft}}$	6	9	1	16
${\mathrm{DCSx}}_{\mathrm{CPPright}}$	7	11	1	19
${\mathrm{DCSx}}_{\mathrm{ICPleft}}$	3	12	1	16
${\mathrm{DCSx}}_{\mathrm{ICPright}}$	11	8	0	19
${\mathrm{COx}}_{\mathrm{MABPleft}}$	6	8	0	14
${\mathrm{COx}}_{\mathrm{MABPright}}$	10	6	2	18
${\mathrm{COx}}_{\mathrm{ICPleft}}$	6	6	2	14
${\mathrm{COx}}_{\mathrm{ICPright}}$	10	7	1	18
${\mathrm{COx}}_{\mathrm{CPPleft}}$	6	6	2	14
${\mathrm{COx}}_{\mathrm{CPPright}}$	9	7	2	18

Due to the hypothesis that CAR assessment reliability is higher when the agreement across indices is higher, an “agreement score” has been provided for each measurement (see Sec. 2). Based on the three categories previously described (high, medium, and mild agreement), among the evaluated measurements (n=20), four have shown high agreement, 14 medium agreement, and two mild agreement. These findings suggest that in a substantial proportion of cases, multiple CAR indices exhibited directionally aligned changes, supporting the robustness of multimodal monitoring. The full results detailed by measurement can be found in Table 7.

With respect to the secondary sub-group analysis to address potential directional redundancies within specific sensor groupings, we have gathered all the results in Table 8.

In brief, within-family analysis has revealed strong internal consistency, but cross-modality alignment with the invasive gold-standard PRx settled near chance-level boundaries. Specifically, DCSx-based indices have demonstrated a balanced, symmetric coupling to pressure reactivity across both cerebral hemispheres, matching the direction of PRx in 53% of cases on the left (9 out of 17 measurement sessions) and 50% on the right (10 out of 20 sessions). Conversely, the COx family has exhibited lower cross-modality alignment with PRx, dropping from 46.7% on the left (7 out of 15 sessions) down to 33.3% within the right hemisphere (6 out of 18 sessions). Further details can be found in the Supplementary Material 1 in Appendix Sec. 6.

## 4 Discussion

In this study, we have analyzed an existing dataset to derive and compare different CAR indices using both standard (invasive) clinical monitors and a combination of those with noninvasive optical neuromonitoring data. We have analyzed both the baseline/stable values of the parameters and their changes (if any) due to a hyperventilation intervention. Due to technical limitations and as CARNet^4^ emphasizes the advantages of time-domain approaches for CAR assessment, we have focused on the traditional analysis method based on the calculation of Pearson’s correlation coefficient in sliding/overlapped temporal windows.^4^ The study was motivated by the reported emerging interest in defining robust CAR indices and measurement protocols both for research and clinical management purposes.^38^

The literature on CAR is extensive and is growing rapidly.^4^^,^^38^^,^^81^^,^^82^ Plenty of potential clinical applications including noninvasive-based modalities have also been explored.^1^^,^^2^^,^^16^^,^^31^^,^^34^^,^^41^^,^^42^^,^^48^^,^^63^^,^^70^^,^^83^^–^^86^^,^^86^^–^^90^ The diversity of techniques utilized to derive CAR across the studies complicates direct comparisons,^17^ so, for this discussion, we have focused on research using noninvasive, time-domain dynamic indices based on Pearson’s correlations^4^^,^^38^ that are consistent with our methods.

Numerous correlation-based indices have been developed to assess CAR,^4^ including PRx,^19^^,^^20^^,^^27^^,^^32^ Mx (between CPP and middle cerebral artery velocity measured via TCD),^25^^,^^27^^,^^73^^,^^89^^,^^91^ Mxa (between ABP and middle cerebral artery velocity via TCD),^6^^,^^92^ Sx (between systolic ABP and cerebral blood flow velocity via TCD),^93^^–^^95^ Dx (between diastolic ABP and cerebral blood flow velocity via TCD),^94^^,^^95^ THx or THI (total hemoglobin reactivity index, derived from total hemoglobin concentration and ABP),^27^^,^^32^^,^^96^ DCSx or BFAx (based on diffuse correlation spectroscopy–measured blood flow),^42^^,^^63^^,^^70^^,^^76^ TOx (tissue oxygen reactivity index from tissue oxygen saturation and ABP),^27^^,^^89^^,^^96^^,^^97^ TOxa (based on near-infrared spectroscopy tissue oxygen index and ABP),^21^ LDx (between laser Doppler–measured CBF and MABP),^98^^,^^99^ HVx (hemoglobin volume index derived from regional total hemoglobin and MABP),^95^ COx,^31^^,^^42^^,^^91^ TDFx (between thermal diffusion flowmetry–measured absolute CBF and CPP),^42^ and ORx (between brain interstitial oxygen tension and CPP).^42^^,^^95^ Overall, these and other indices may appear the same/similar, but a detailed look at the modalities that are utilized reveals that they are sensitive to different aspects of how CAR impresses itself on different spatio-temporal scales and biological/physiological processes that are found in the brain.

In our analysis, we have focused on PRx, DCSx, and COx, with modifications in the latter two through alternative signal sources (research devices versus clinical monitors; different, more complex implementation of modalities compared with widely available methods). PRx analysis is comparable to the above-cited references and is one of the most common methods because it utilizes standard clinical neuromonitors. However, PRx does not directly follow the primary hypothesis of CAR—i.e., the regulation of cerebral blood flow to keep it constant with respect to changes in MABP or its regulation to meet the demand.

DCSx and COx, on the other hand, look at two different aspects—oxygen availability and tissue perfusion. It is important to note that, to date, DCSx has always been measured by research devices that estimate microvascular CBF. There are other methods that estimate either macrovascular or very-localized (i.e., $∼1\;{\mathrm{mm}}^{3}$ volume) microvascular CBF—some with invasive monitors that may or may not agree with DCSx results. On the other hand, COx, i.e., NIR spectroscopy (NIRS) derived CAR index, has often been derived using clinical, cerebral oximeters.^29^^,^^30^^,^^32^^,^^100^^,^^101^ In our case, we have utilized a more complex yet presumably more accurate and precise, research-grade NIRS system—based on TRS.^43^ This provides potential for superior depth discrimination if a more complex analysis method (tackling systematic issues it may raise) is validated in the future. This could eventually provide improved cerebral selectivity compared with traditional continuous-wave NIRS and ensures that these measurements reflect cortical hemoglobin saturation with reduced extracerebral contamination.^43^^,^^44^^,^^102^^–^^104^ In both cases, for completeness, we have used ICP, CPP, and MABP in the analysis. These aspects should be kept in mind when comparing our results to the literature.

In Fig. 1, we have shown representative time traces that highlight the main features of this work. There is a rich dataset that allows us to compare invasive measures with noninvasive measures and use them collectively to derive a variety of CAR indices. We see that although different CAR indices show similar results in some subjects, they differ in others. Overall, we have observed decreased median values in ${\mathrm{PaCO}}_{2}$ and ICP and increased MABP following a period of HV, suggesting a beneficial physiological response. This is not a new result and has been previously published^105^^–^^108^ and is in agreement with the literature. However, CPP has presented a significant increase, in agreement with Refs. 108 and 109 while in contrast with Ref. 53. CAR indices have responded heterogeneously. The group level (Fig. 3), the only index to demonstrate a robust statistical decrease during recovery, after accounting for multiple comparisons, has been ${\mathrm{DCSx}}_{\mathrm{ICPleft}}$ ($p_{\text{adjusted}}=0.030$). Other indices, such as ${\mathrm{DCSx}}_{\mathrm{MABPleft}}$, which previously exhibited significant decreases in uncorrected analyses ($p_{\text{raw}}=0.019$), have not survived the FDR adjustment ($p_{\text{adjusted}}=0.129$). Although this indicates a notable downward trend across several subjects, the high inter-subject variability inherent in a TBI population prevents these trends from reaching group-level significance once corrected. This statistical outcome underscores the importance of our single-subject evaluation (Table 6).

There is no surprise here that the results are heterogeneous. Both clinical experience of the clinicians when managing these subjects and the literature^22^ highlight this. Furthermore, methodological variations contribute to this heterogeneity, for example, Briggs et al.^23^ highlighted that averaging strategies (i.e., heartbeat versus second-level) and correlation window lengths affect CAR index sensitivity and inter-individual variability. However, heartbeat-level averaging, while potentially reducing noise, departs from the conventional low-frequency (<0.05  Hz) focus of CAR. Moreover, uncertainty remains about whether different CAR indices capture the same physiological regulatory processes as discussed above.^1^ These indices measure correlations between ABP or CPP and downstream signals like ICP, CBFV, or ${\mathrm{rSO}}_{2}$, which may also reflect seizures, edema, or metabolic changes, contributing to inter-index variability.

This is further supported by physiology. For example, Brassard et al.^17^ noted that different stimuli (i.e., spontaneous versus induced ABP fluctuations) may activate distinct autoregulatory mechanisms. This limits index comparability and cross-method generalizations, as weak inter-index correlations suggest each captures different aspects of CAR. PRx is invasive; NIRS-based indices are noninvasive but prone to individual-specific confounders, as explained in the previous paragraph. No single index is universally the best; a multimodal approach and a detailed contextual interpretation are essential.^1^ This study provides an example of how such data can be obtained with relatively low-cost, bedside systems. It is worth noting that CAR’s response to HV also remains poorly understood.^110^ HV reduces ${\mathrm{PaCO}}_{2}$ and ICP through vasoconstriction, as we have also found (Sec. 3.3, initial paragraph).

Our results have shown a heterogeneous distribution of trends across the evaluated CAR indices, as reported in Tables 6 and 7, similarly to other studies.^16^^,^^27^^,^^28^^,^^32^^,^^42^^,^^70^^,^^90^^,^^111^^–^^114^ Highton et al.^27^ suggested that different frequencies and signal properties across modalities limit correlation. Although ICP, TCD, and NIRS share low-frequency content, their spectral differences may impair alignment, affecting correlation-based indices. Baker et al.^42^ attributed poor cross-index agreement to DCS limitations in measuring absolute CBF. Liu et al.^112^ emphasized the role of exogenous noise, reporting intra-patient inconsistencies over time. CAR indices are also constrained by modality-specific errors: technological (i.e., sensitivity to noise)^39^ and physiological (i.e., micro- versus macrovascular sources).^9^^,^^28^^,^^31^^,^^32^^,^^114^ The distinct behavior observed between DCSx-based and COx-based hemispheric agreement score sub-groups highlights that the composite “agreement score” does not merely reflect a redundant consensus of tightly coupled sensors. Instead, it serves as a regional and systemic quantifier of physiological synchronicity, faceting CAR response across different vascular beds. Interestingly, the lowest cross-modality agreement has occurred between PRx, with the invasive ICP sensor generally inserted on the right side, and the right-sided oxygenation family (${\mathrm{COx}}_{\text{right}}$ at 33.3%). Our findings seem to suggest that although DCSx tracks global perfusion pressure surrogates relatively symmetrically, COx undergoes a certain level of local decoupling at the primary site of injury and monitoring, where acute TBI pathologies have frequently driven severe microvascular congestion, localized edema, and disrupted metabolic buffering. This ultimately demonstrates the clinical necessity of capturing localized, multimodal hemispheric profiles alongside global macrovascular metrics.

Elting et al.^9^ noted that deoxyhemoglobin lags behind oxyhemoglobin due to capillary transit and washout, which we have not corrected for. These delays occur on a seconds scale, shorter than our 300-s correlation windows, and thus unlikely to bias index computation, but may still affect signal alignment in shorter windows. PRx may better reflect elevated ICP, whereas COx may indicate perfusion risks.^114^ Bird et al.^113^ have found that COx may conflict with other indices due to issues with ${\mathrm{StO}}_{2}$ signals, which may remain normal in ischemic states due to impaired oxygen diffusion or metabolism. Bogli et al.^2^ found COx often yielded correlations below 0.3, likely due to device smoothing and low-resolution steps (1%), identifying COx as a notable outlier across multiple analyses. In line with these findings, in our study, COx distributions have shown no significant changes during HV and remained tightly clustered around zero. Although low variability in correlation indices can sometimes be misinterpreted as a lack of signal variance, our results suggest this reflects preserved autoregulatory stability rather than technical limitations. In fact, the spectral analysis of the ${\mathrm{StO}}_{2}$ signal revealed robust physio-logical oscillations in the 0.003 to 0.05 Hz band. A separate time-frequency decomposition (example in the Supplementary Material 3 in Appendix Sec. 6) also confirms that this low-frequency power is nonstationary and spans a narrow but finite and fluctuating (in power and frequency) bandwidth corresponding to known endothelial, neurogenic and, in some instances, myogenic vasomotion, rather than static single-frequency hardware noise. Furthermore, we have verified that the ${\mathrm{StO}}_{2}$ signal exhibited a coefficient of variation (CV 4.3% to 7.6%) statistically comparable to that of BFI (CV 7.5% to 10.2%), aligning with established TRS literature^115^^–^^117^. The observed stability of COx may therefore signal a protective physiological effect where oxygen delivery and consumption remain balanced despite HV-induced flow reductions. Future work on both healthy and patients with cerebral pathologies during rest can be carried out to evaluate different frequency content in the signals, including BFI.

Despite this, a substantial number of sessions in our study have shown concordant directional trends across multiple indices. Using our “agreement score” categories, we have observed that 4/20 measurements showed high, 14 medium, and 2 mild score (see Table 7 in the Supplementary Material in Appendix Sec. 6.1), supporting the value of looking into multimodal convergence for reliability.

In fact, a recent Delphi consensus survey highlights concerns about the accuracy and reliability of CAR indices and advocate for integrating uncertainty metrics to improve interpretability, robustness, and confidence in their use.^2^^,^^7^^,^^40^ For instance, high-frequency fluctuations in PRx may reflect methodological noise rather than physiological change,^7^ underscoring the need to distinguish signal artifact from genuine autoregulatory behavior. We believe that the metrics we utilize with the methods we introduce take a step toward this end. In our cohort, COx indices, especially from the left hemisphere during recovery, have shown less variability than PRx (see Fig. 5), as measured by IQR, suggesting some indices are more robust to artifacts. We have also explored inter-index correlation as a marker of reliability (refer to Tables 7 and 6), although in a simplified manner. Bogli et al.^1^ showed that indices from different signals (i.e., ICP, CBF, NIRS-based signals) correlate more during physiologically stable periods. Such convergence likely reflects true CAR behavior. However, shared noise or artifacts could also increase inter-index correlation. Therefore, poor intercorrelation in such periods of low signal quality may not indicate true physiological disagreement.

Given our limited sample (n=20 when all measurements are available for a parameter, while n<20 otherwise, with min=14), we have performed within-measurement comparisons to assess HV effects. Most significant changes reflected decreased CAR indices ipsilateral to the invasive ICP sensor (see Tables 6 and 7), suggesting improved CAR. This analysis has revealed/confirmed several things: (a) different indices may provide different results on an individual, (b) combination of different indices may provide a more robust assessment (to be verified), and (c) the personalization of the management based on CAR requires careful considerations about the utilized modality.

Some authors^75^^,^^98^ advise computing CAR indices only when MABP fluctuates >2  mmHg to avoid unreliable near-zero correlations caused by low signal variance. In our dataset, only one session failed this threshold, and we have avoided these strict criteria. Similarly, we have found that all subjects, including the single case in our cohort with a craniectomy, exhibited ICP variability above  >2  mmHg in all but four baseline sessions (between 0.5 to 2 mmHg), whereas no threshold value for ICP has been found in the existing literature. Bogli et al.^1^ also proposed quality control filters, such as physiological plausibility ranges for ABP and ICP. We have not applied all filters, but we emphasize the importance of data curation, and have applied strict rules when utilizing the optical data^48^ (Sec. 2).

In addition, we have not applied explicit temporal filtering beyond the moving-window correlation inherent to the time-domain approach. However, the re-binning of all signals into 5-s intervals implicitly constrained the frequency content of the analysis: the resulting sampling rate (0.2 Hz) limits representable frequencies to below 0.1 Hz, consistent with the slow-wave band relevant for dCAR assessment. This choice ensured standardized sampling across modalities. Although this approach aligns with CARNet recommendations for time-domain analysis, it differs from the method proposed by Kostoglou et al.,^4^ who explicitly remove the DC (0 Hz) component using high-pass filtering.

A primary limitation of this study is the small sample size, which has restricted our ability to carry out substantial group-level analyses. For example, as we could not standardize the time of the measurement across subjects, our variability is higher,^3^ which, in turn, requires more patients.

We acknowledge the technical trade-offs inherent in using semi-infinite homogeneous models for optical measurements. Although this model is a standard approximation that relies on the condition that the medium is scattering-dominant ($\mu_{s}^{\prime }\gg \mu_{a}$), it does not explicitly account for the distinct layers of the extracerebral tissue. Although we do not employ explicit temporal gating, the time-resolved nature of our TRS system offers fundamentally higher sensitivity to deeper tissues than CW-NIRS methods. In fact, unlike the latter, which provides a single intensity-integrated value, TRS captures the full distribution of photon arrival times (DTOF). ${\mathrm{StO}}_{2}$ has been calculated through a two-step retrieval of optical properties: initial parameter estimation via the moments method^118^^–^^120^ followed by a nonlinear least-squares fit to the DTOF curves using a semi-infinite homogeneous diffusion model. The higher-order moments of the DTOF, specifically the variance, are mathematically more sensitive to deep-layer absorption and less influenced by superficial scattering changes. By fitting the DTOF using the moments method as an initial step, we effectively leverage this temporal information to retrieve cerebral optical properties with superior depth discrimination compared with CW-based systems. Similarly, for DCS, although a 2.5 cm separation may include extracerebral signals, recent literature and simulations support that the resulting BFI remains highly sensitive to cerebral changes^121^^–^^127^. Moreover, our DCS measurements provide BFI by standard fitting utilizing TRS-derived optical properties as inputs. Therefore, although these models do not explicitly account for tissue heterogeneity, our implementation provides enhanced depth discrimination and reduces the influence of superficial extracerebral tissues on both ${\mathrm{StO}}_{2}$ and BFI estimations. Future studies incorporating multilayer or anatomical models could further refine these quantifications.

In our interpretation, we have not considered secondary cerebral events frequently seen in TBI, such as B-waves, spreading depolarizations, or plateau waves, which can influence CAR dynamics.^2^^,^^27^ Plateau waves, in particular, reflect complex interactions between CBF, autoregulation, and intracranial compliance and may alter ICP-derived metrics during sustained elevations. This information is available for part of the dataset, but given the small sample size, it would not be generalizable.

Factors such as age, sex, and comorbidities also influence CAR^16^ and may influence different physiological signals utilized for calculating CAR in a different manner. This highlights the main point of the study—different CAR indices may give different results—but cannot be utilized to correct for the changes, again due to relatively small sample size.

Although we present hemispheric data, 88% of our cohort had the ICP bolt located in the right hemisphere. Only one patient presented a configuration where the sensor was contralateral to a focal lesion. Given this homogeneity, the current hemispheric designation effectively captures the relationship between focal and global CAR for the vast majority of the subjects without re-grouping the sample.

No healthy control group has been included. Although desirable for comparison, the invasiveness of ICP monitoring renders such a control group ethically unfeasible; fully noninvasive approaches would be needed for future studies. This is a barrier to most CAR-related studies in the literature: the technologies are tested on pathological scenarios.

In addition, our analysis does not account for the phase difference between cerebral blood flow velocity and ABP, which has been shown to vary with oscillation frequency—typically increasing at 0.05 Hz compared with 0.1 Hz.^17^ This could be evaluated in a dedicated study with a measurement of macrovascular, i.e., TCD, measurement of CBF alongside DCS.

Finally, the cohort consisted exclusively of TBI patients undergoing invasive monitoring. Although this limits generalizability, TBI remains the primary context for CAR monitoring and the patient population most likely to benefit from its clinical application.^1^

### 4.1 Future Directions and Implementations

By leveraging newly developed, reliable noninvasive monitors for MABP, fully noninvasive CAR assessments may soon become feasible. This is especially promising given recent advances in machine learning, which allows estimation of ICP from BFI pulsatility features.^128^^–^^131^

Future studies with larger cohorts may help define reliable thresholds to differentiate between intact and impaired CAR, as established for TCD-based methods. Potential approaches include percentile-based criteria, ROC analysis, or empirical identification of critical closing pressure, where the regression slope between ABP and CBF reaches zero.

The novelty of utilizing ICP-derived indices, such as the correlation between ICP and flow/oxygenation, lies in their ability to characterize ICP dynamics independent of systemic blood pressure. Traditional indices like Mx rely on MABP, yet in several clinical populations, average ICP and MABP values often remain within physiological norms, such as in some types of hydrocephalus and related CSF disorders (i.e., in idiopathic normal pressure hydrocephalus). In these "normal" cases, the mean value provides little diagnostic or prognostic value. However, the dynamic relationship between ICP fluctuations and cerebral flow acts as a proxy for the brain’s compensatory reserve and venous outflow resistance. This is analogous to the FIx index, which identifies a loss of synchrony between flow and pressure as a marker of vascular exhaustion. From a clinical end-user perspective, these insights are vital. For instance, clinicians frequently utilize the morphology and synchrony of ICP waves to determine surgical candidacy for shunting. Furthermore, these indices offer a potential pathway for future entirely noninvasive monitoring using noninvasive diffuse optical methods.

Routine dCAR monitoring using user-friendly, continuous, real-time systems is essential for individualized therapy, secondary injury prevention, and therapeutic window identification following TBI.^3^^,^^34^ Currently, ICM+, a research software platform, partially addresses this need by providing real-time PRx-based CAR estimates,^78^^,^^111^^,^^132^^,^^133^ although it is not yet licensed as a medical device. Since 2024, Edwards Lifesciences^134^ commercializes an FDA-cleared monitor, the HemoSphere Alta^TM^ Monitor, that provides the so-called cerebral autoregulation index. Its utility is under debate.

In principle, multimodal CAR indices could inform autoregulation-oriented management strategies, moving beyond U-shaped representations of PRx and CPP toward fully personalized care.^23^^,^^24^^,^^135^ Incorporating real-time uncertainty estimates into CAR indices would support more confident clinical decisions, particularly when uncertainty is low.^7^ The expanding field of neurocritical care informatics is key to integrating and visualizing multimodal data for bedside use. However, implementation in nonspecialized settings remains a challenge. Recently developed devices aim to address this by simplifying outputs and providing targeted training.^2^^,^^136^

Finally, the interaction between CAR and misery perfusion represents an important area for future study. These phenomena may influence each other through opposing yet plausible mechanisms, meriting further exploration.^48^^,^^137^

## 5 Conclusion

In conclusion, we have utilized different ways of measuring dCAR from the same subject at the same time and have shown that their interpretation requires care. Although PRx remains the established clinical gold standard for dCAR evaluation, the addition of synchronized optical indices allows for a multimodal cross-validation at the bedside. The behavior of these indices can diverge depending on the systemic driver and the physiological variable being monitored, whether it be microvascular BFI or ${\mathrm{StO}}_{2}$. The value of hybrid diffuse optical monitors lies in their ability to provide these two complementary perspectives simultaneously, which, when integrated with invasive measurements like MABP or ICP, offers a more comprehensive assessment of the autoregulatory status. We argue that this integrated approach improves the robustness of results for the individual patient by resolving ambiguities that may arise when relying on a single parameter once validated across both clinical and healthy populations. Ultimately, our findings suggest that clinical thresholds should be tailored to the specific modalities utilized, establishing hybrid devices as sophisticated tools for personalized theragnostics in complex clinical scenarios, such as in populations with hypertension, stroke, or spinal cord injury.

## 6 Appendix: Supplementary Material

### 6.1 Supplementary Material 1

**Fig. 6:**
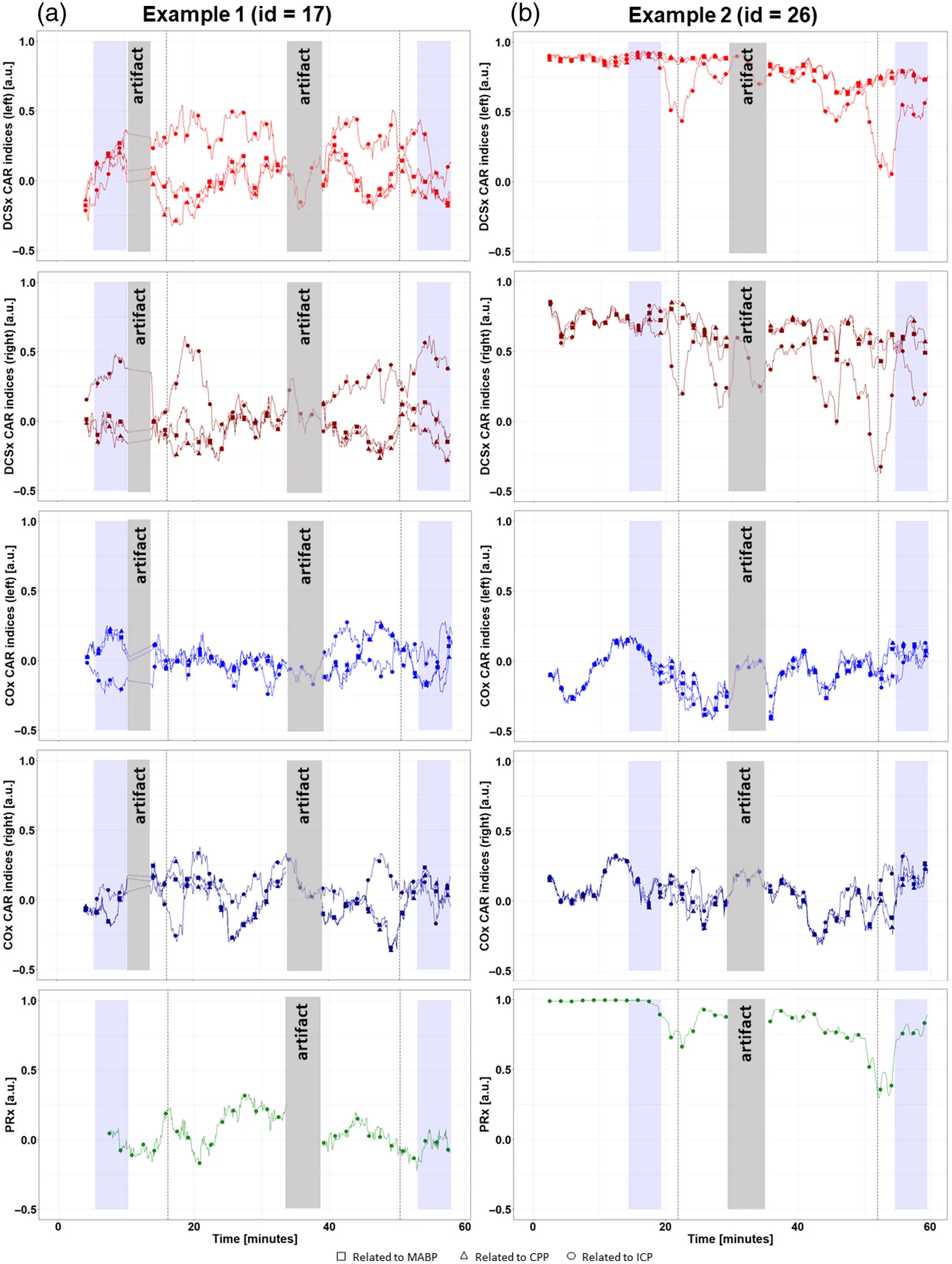
Detailed time-traces of cerebral autoregulation indices (see Fig. 1) separated into distinct subplots. Two clinical cases are presented for comparison: Example 1 (a) and Example 2 (b). Vertical dashed lines delineate the transition between baseline (HV beginning) and recovery (HV end) periods. Light-blue shaded areas indicate the 5-min segments selected for steady-state analysis, whereas gray areas denote identified artifacts. DCSx-related indices (red shades) are calculated from BFI, separated for the left (first row) and right (second row) cerebral hemispheres. COx-related indices (blue shades) are calculated from ${\mathrm{StO}}_{2}$, separated for the left (third row) and right (fourth row) cerebral hemispheres. PRx index (green) is reported in the bottom row), providing a global comparison across the different monitoring modalities. Consistent with the naming conventions, left-sided indices are represented by lighter lines/markers, while right-sided indices use darker shades. Marker shapes differentiate the systemic driver used for the correlation: squares for MABP, triangles for CPP and circles for ICP. HV, hyperventilation; MABP, mean arterial blood pressure; CPP, cerebral perfusion pressure; ICP, intracranial pressure; PRx, pressure reactivity index; DCSx, CAR index calculated from blood flow index (BFI); COx, CAR index calculated from tissue oxygen saturation (${\mathrm{StO}}_{2}$).

Here, we report in Fig. 6 the time-traces related to DCSx and COx in a different fashion than in Fig. 1, to better appreciate their temporal evolution.

Table 7 gathers all the results obtained by Wilcoxon tests to find significant differences between recovery and baseline. Significant increases are represented by upward arrows, significant decreases by downward arrows, nonsignificant changes by dashes and when measurement where not present or discarded, the entry contains NA. The last column gather the “agreement score” as a percentage out of the total available measurements.

**Table 7:** Results of the significant changes for all variables and “agreement score’ among the indices for each measurement. Significant increases are represented by upward arrows, significant decreases by downward arrow, nonsignificant changes by dashes, and when measurement where not present or discarded the entry contains NA.

id	PRx	${\mathrm{DCSx}}_{\mathrm{MABPleft}}$	${\mathrm{DCSx}}_{\mathrm{MABPright}}$	${\mathrm{DCSx}}_{\mathrm{CPPleft}}$	${\mathrm{DCSx}}_{\mathrm{CPPright}}$	${\mathrm{DCSx}}_{\mathrm{ICPleft}}$	${\mathrm{DCSx}}_{\mathrm{ICPright}}$	${\mathrm{COx}}_{\mathrm{MABPleft}}$	${\mathrm{COx}}_{\mathrm{MABPright}}$	${\mathrm{COx}}_{\mathrm{CPPleft}}$	${\mathrm{COx}}_{\mathrm{CPPright}}$	${\mathrm{COx}}_{\mathrm{ICPleft}}$	${\mathrm{COx}}_{\mathrm{ICPright}}$	agreementscore [%]
8	↑	↓	NA	↓	—	↓	NA	↓	↓	↓	↓	↓	↑	81.8
9	↑	—	—	↓	↓	↑	↑	↑	↑	—	—	↑	↑	75
11	↓	↓	↑	↓	↓	↑	↑	NA	↑	↑	—	↑	NA	63.6
12	↓	↓	↓	↓	↓	↓	↓	NA	—	NA	↑	NA	↓	80
13	↓	↑	↑	↑	↑	↓	↑	↑	↑	↑	↑	—	↓	75
17	↓	↓	↑	↓	↓	—	↑	↓	↑	↓	↑	↑	—	45.5
18	↑	↑	↑	↑	↑	↑	↓	↓	↑	↑	↓	↓	↑	63.6
19	↓	↑	↑	↑	↑	↓	↓	↓	↓	↓	↓	↑	↑	50
21	↓	NA	↑	NA	↓	NA	↑	↑	NA	↑	NA	↓	NA	66.7
22	↓	NA	↓	NA	↓	NA	↓	NA	↑	NA	—	NA	↑	57.1
23	↑	NA	↑	NA	↑	NA	↑	NA	↓	NA	↓	NA	↓	50
24	↑	↓	↑	↑	↓	↓	↑	↑	↓	↑	↑	↓	↓	50
26	↓	↓	↓	↓	—	↓	↓	↓	↑	↓	↑	–	↑	66.7
27	↓	↓	↑	↓	↑	↓	↑	NA	↑	NA	↑	NA	↓	60
28	↓	NA	↓	NA	↓	NA	↑	NA	NA	NA	NA	NA	NA	66.7
29	↑	↓	↓	↓	↓	↓	↑	↓	—	↓	↓	↑	↑	63.6
30	↓	↓	↓	↓	↓	↓	↓	↑	↑	—	↑	↑	↓	63.6
31	↓	↓	—	↑	↑	↓	↓	↓	↓	↑	↓	↓	↑	63.6
32	↑	↓	↑	—	↑	↓	↓	↑	↑	↑	↑	↓	↓	45.5
33	↑	↑	↓	↑	↓	↓	↑	↓	↓	↓	↓	↑	↑	50

To address potential confounding variables related to regional redundancy or uneven sensor weights in the “agreement score,” Table 8 presents an interleaved sub-group analysis according to family (DCSx versus COx) and cerebral hemisphere (left versus right) agreement scores (AS) and directional changes (dir). Optical hemispheric clusters have also been considered. This breakdown isolates the structural consensus trends, validating the presence of localized physiological heterogeneity. Once compared with the original “agreement score” and PRx trend, these have revealed: (1) a pronounced decoupling between local COx-based families and PRx, particularly within the right hemisphere where agreement dropped to 33% (6 out of 18 measurement sessions), whereas to 46.7% for the left (7 out of 15 measurement sessions). This regional divergence can be tied to probe positioning: for the vast majority of our cohort, the invasive ICP sensor used to compute PRx was localized to the right cerebral hemisphere; (2) DCSx-based indices maintain a moderate baseline alignment with PRx across both hemispheres ( ∼50% to 53%); (3) when combining the optical modalities to evaluate purely regional hemispheric behavior (combining DCSx and COx parameters into a single consensus), the pooled “Optical left” consensus has matched the direction of the global macrovascular PRx in 53.0% of monitored sessions (9 out of 17), whereas the pooled “Optical right” consensus has demonstrated a 50.0% alignment rate with PRx (10 out of 20 sessions). This intermediate, chance-level alignment across both hemispheres confirms that a regional optical consensus tracks systemic pressure drivers independently, avoiding skewed inflation while masking finer cross-modality conflicts within the hemisphere itself.

**Table 8:** Breakdown of local consensus directions and family-wise sub-group agreement scores across hemispheres. The initial eight columns following the one containing the measurement “id” represent the dominant directional trend (*dir*) and corresponding intra-family agreement score (*AS* [%]) calculated independently for the left flow family (${\mathrm{DCSx}}_{L}$), right flow family (${\mathrm{DCSx}}_{R}$), left oxygenation family (${\mathrm{COx}}_{L}$), and right oxygenation family (${\mathrm{COx}}_{R}$). The following four columns quantify the explicit cross-modality alignment within the same hemisphere for the optical methods only (“Opt”). The last two columns are copied from Table 7, reporting PRx and the original “agreement score” for comparison reasons. Direction indices mark significant trends (↑ and ↓), unvarying responses (−), or omitted configurations (NA).

id	${\mathrm{DCSx}}_{\mathrm{Ldir}}$	${\mathrm{DCSx}}_{\mathrm{LAS}}[\%]$	${\mathrm{DCSx}}_{\mathrm{Rdir}}$	${\mathrm{DCSx}}_{\mathrm{RAS}}[\%]$	${\mathrm{COx}}_{\mathrm{Ldir}}$	${\mathrm{COx}}_{\mathrm{LAS}}[\%]$	${\mathrm{COx}}_{\mathrm{Rdir}}$	${\mathrm{COx}}_{\mathrm{RAS}}[\%]$	${\text{Optical}}_{\mathrm{Ldir}}$	${\text{Optical}}_{\mathrm{LAS}}[\%]$	${\text{Optical}}_{\mathrm{Rdir}}$	${\text{Optical}}_{\mathrm{RAS}}[\%]$	${\mathrm{PRx}}_{\mathrm{dir}}$	Agreementscore [%]
8	↓	100.0	−	100.0	↓	100.0	↓	66.7	↓	100.0	↓	66.7	↑	81.8
9	↓	33.3	↓	33.3	↑	66.7	↑	66.7	↑	50.0	↑	50.0	↑	75.0
11	↓	66.7	↑	66.7	↑	66.7	↑	33.3	↑	60.0	↑	50.0	↓	63.6
12	↓	100.0	↓	100.0	NA	NA	↓	33.3	↓	50.0	↓	66.7	↓	80.0
13	↑	66.7	↑	100.0	↑	66.7	↑	66.7	↑	66.7	↑	83.3	↓	75.0
17	↓	66.7	↑	66.7	↓	66.7	↑	66.7	↓	83.3	↑	66.7	↓	45.5
18	↑	100.0	↑	66.7	↓	66.7	↑	66.7	↑	83.3	↑	66.7	↑	63.6
19	↑	66.7	↑	66.7	↓	66.7	↓	66.7	↓	50.0	↓	50.0	↓	50.0
21	NA	NA	↑	66.7	↑	66.7	NA	NA	↑	66.7	↑	66.7	↓	66.7
22	NA	NA	↓	100.0	NA	NA	↑	66.7	NA	NA	↓	50.0	↓	57.1
23	NA	NA	↑	100.0	NA	NA	↓	100.0	NA	NA	↓	50.0	↑	50.0
24	↓	66.7	↑	66.7	↑	66.7	↓	66.7	↓	50.0	↑	50.0	↑	50.0
26	↓	100.0	↓	66.7	↓	66.7	↑	100.0	↓	83.3	↑	50.0	↓	66.7
27	↓	100.0	↑	100.0	NA	NA	↑	66.7	↓	50	↑	83.3	↓	60.0
28	NA	NA	↓	66.7	NA	NA	NA	NA	NA	NA	↓	66.7	↓	66.7
29	↓	100.0	↓	66.7	↓	66.7	↓	33.3	↓	83.3	↓	50.0	↑	63.6
30	↓	100.0	↓	100.0	↑	66.7	↑	66.7	↓	50.0	↓	66.7	↓	63.6
31	↓	66.7	↓	33.3	↓	66.7	↓	66.7	↓	66.7	↓	50.0	↓	63.6
32	↓	66.7	↑	66.7	↑	66.7	↑	66.7	↓	50.0	↑	66.7	↑	45.5
33	↑	66.7	↓	66.7	↓	66.7	↓	66.7	↓	50.0	↓	66.7	↑	50.0

### 6.2 Supplementary Material 2

Detailed theoretical foundations, mathematical derivations, and coefficients for ${\mathrm{StO}}_{2}$ and BFI retrieval are provided in Ref. 59; we summarize the salient points here for completeness.

${\mathrm{StO}}_{2}$ has been determined through a two-step analysis of the TRS distribution of photon arrival times curves to ensure high accuracy and depth selectivity. First, the moments method has been applied to obtain initial estimates of the absorption ($\mu_{a}$) and reduced scattering ($\mu_{s}^{\prime }$) coefficients.^118^^–^^120^ This method allows for the direct correction of the mean time of flight and variance by subtracting the respective moments of the instrument response function (IRF), a technique that enhances sensitivity to deeper cortical information. Second, these coefficients served as the optimized initial guesses for a nonlinear least-squares fitting procedure, where the analytical solution for a semi-infinite homogeneous medium has been convoluted with the IRF to minimize the $\chi^{2}$ distance from experimental data. $\mu_{s}^{\prime }$ and absorption $\mu_{a}$ coefficients have been retrieved at 687 and 830 nm without water or other chromophore corrections, as these two wavelengths are sufficient to characterize the primary hemoglobin species in this range. Using $\mu_{a}$ s measured at 687 and 830 nm, we have solved a linear system of two equations to independently calculate the concentrations of oxy- and deoxy-hemoglobin via the Beer–Lambert law, using compiled molecular extinction coefficients according to the literature.^138^ As oxy- and deoxy-hemoglobin concentrations are the principal absorbers in the near-infrared optical window, other absorbers have been discarded to simplify the linear system. This dual-wavelength approach is a standard validated method for characterizing primary hemoglobin species in the near-infrared window without needing corrections for water or other minor chromophores. ${\mathrm{StO}}_{2}$ has been subsequently derived from these absolute concentrations. A comprehensive description of the data analysis pipeline, including IRF subtraction, background correction, and fitting regions of interest, can be found in Ref. 59.

DCS measurements have been used to retrieve the BFI by fitting the autocorrelation function to the analytical solution of the correlation diffusion equation, utilizing the TRS-derived optical properties to improve fit accuracy.

### 6.3 Supplementary Material 3

**Fig. 7:**
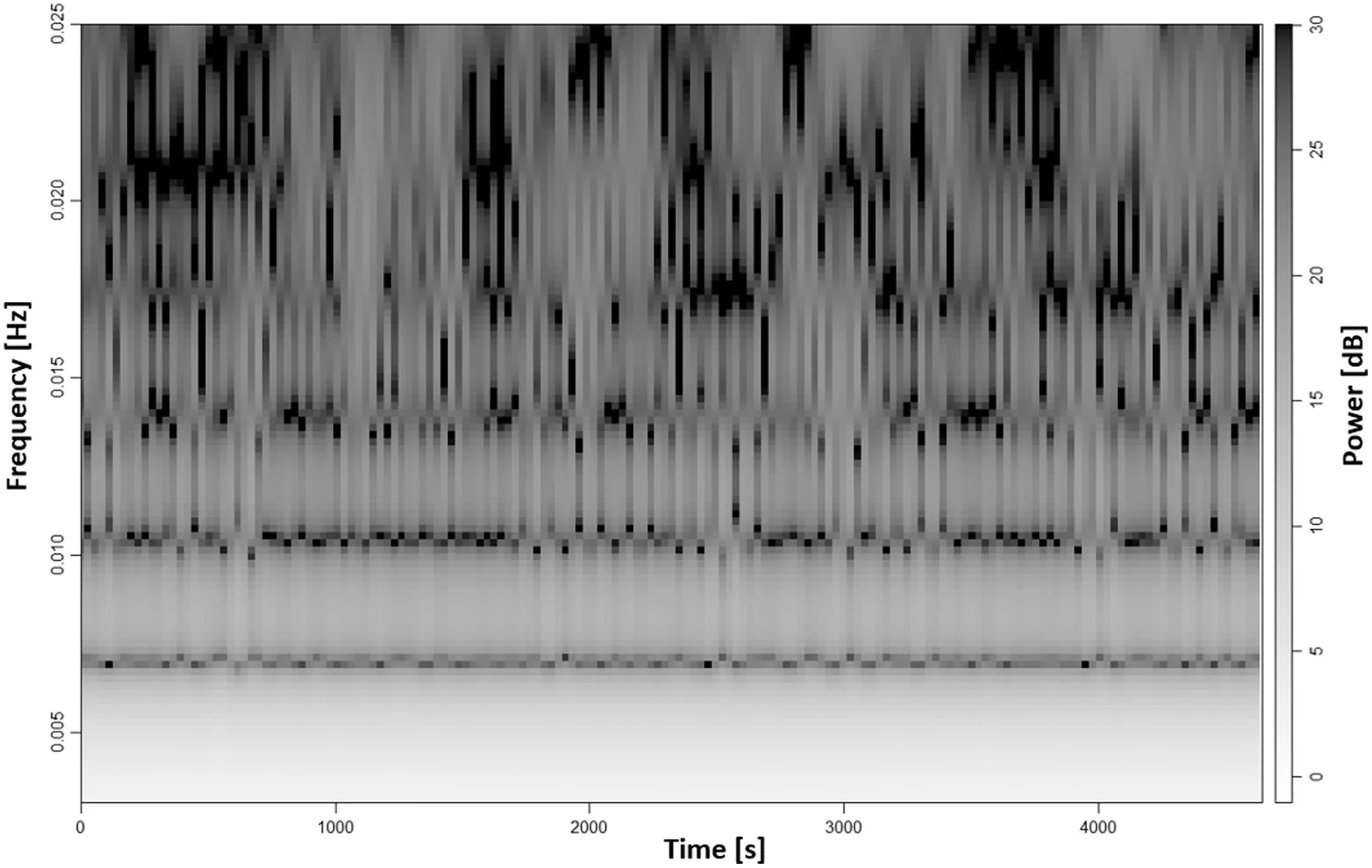
Representative time-frequency analysis in the 0.003 and 0.025 Hz range showing oscillations that correspond to endothelial, neurogenic and myogenic bands.

Figure 7 shows a representative time-frequency analysis for Example 2 (id=26, right cerebral hemisphere) from Fig. 1 (main text), spanning the 0.003 to 0.025 Hz range. The analysis shows oscillations corresponding to endothelial, neurogenic, and myogenic frequency bands. This range was selected to enhance the visualization of the identified frequency peaks and their temporal variability, although a wider range up to 0.05 Hz was also explored (data not shown).

## Data Availability

Data are available in the Zenodo repository at https://doi.org/10.5281/zenodo.22133950.
